# Tissue-specific inflammation induces cell state plasticity with oncogenic addiction in mucosal melanoma

**DOI:** 10.1126/sciadv.ady4536

**Published:** 2026-04-01

**Authors:** Xuhui Ma, Yanni Ma, Li Zhang, Ruixin Liu, Ronghui Xia, Meiling Hao, Xiaole Song, Yinan Chen, Yang Zheng, Hao Wang, Hao Luo, Shengnan Zheng, Jie Yang, Qin Yang, Ruixin Jiang, Xiangyu Chen, Pengcong Hou, Kaiyuan Hui, Qian Bian, Bin Jiang, Xiaodong Jiang, Min Jiang, Yanjie Zhang, A. Hunter Shain, Guoxin Ren, Ming Lei, Robert L. Judson-Torres, Wei Guo, Hanlin Zeng

**Affiliations:** ^1^ Department of Oral and Maxillofacial-Head and Neck Oncology, Shanghai Ninth People’s Hospital, Shanghai Jiao Tong University School of Medicine, Shanghai 200011, China.; ^2^ College of Stomatology, Shanghai Jiao Tong University, Shanghai 200025, China.; ^3^ Shanghai Key Laboratory of Stomatology & Shanghai Research Institute of Stomatology, National Clinical Research Center of Stomatology, Shanghai 200011, China.; ^4^ Department of Oncology, Shanghai Ninth People’s Hospital, Shanghai Jiao Tong University School of Medicine, Shanghai 200011, China.; ^5^ Shanghai Institute of Precision Medicine, Shanghai Ninth People’s Hospital, Shanghai Jiao Tong University School of Medicine, Shanghai 200011, China.; ^6^ Shanghai Institute of Immunology, Department of Immunology and Microbiology, Shanghai Jiao Tong University School of Medicine, Shanghai 200011, China.; ^7^ Department of Oral Pathology, Shanghai Ninth People’s Hospital, Shanghai Jiao Tong University School of Medicine, Shanghai 200011, China.; ^8^ Department of Otolaryngology, Eye & ENT Hospital, Fudan University, Shanghai 200031, China.; ^9^ Mucosal Melanoma Diagnosis and Treatment Center, Eye & ENT Hospital, Fudan University, Shanghai 200031, China.; ^10^ Department of Plastic and Reconstructive Surgery, Shanghai Ninth People’s Hospital, Shanghai Jiao Tong University School of Medicine, Shanghai 200011, China.; ^11^ Department of Oncology, The Affiliated Lianyungang Hospital of Xuzhou Medical University, Jiangsu 222061, China.; ^12^ Department of Oncology, The First Affiliated Hospital of Soochow University, Suzhou 215006, China.; ^13^ Department of Dermatology, University of California, San Francisco, San Francisco, CA 94158, USA.; ^14^ Helen Diller Family Comprehensive Cancer Center, University of California, San Francisco, San Francisco, CA 94158, USA.; ^15^ Department of Dermatology, University of Utah Salt Lake City, UT 84112, USA.; ^16^ Huntsman Cancer Institute, Salt Lake City, UT 84112, USA.

## Abstract

Mucosal melanoma (MM), an aggressive melanoma subtype arising in mucosal tissues, displays resistance to therapies effective in cutaneous melanoma. To understand how mucosal microenvironment contributes to treatment nonresponsiveness, we performed integrative analysis of single-cell and bulk messenger RNA sequencing data derived from oral mucosa–originated melanoma and revealed that mucosa-specific inflammation induces enrichment of low-pigmented neural crest–like cancer cell, mediated by COX2^+^ macrophages and their secretome. Maintenance of this inflammation-induced neural crest–like state in cancer cells depends on HER2 and HER3 activation. Inhibition of HER2/3 by pan-HER inhibitors blocks cell state plasticity and overcomes chemoresistance in primary MM cell lines and patient-derived xenograft (PDX) models. These findings provide insights into how the tissue of origin determines cancer aggressiveness, highlight the role of mucosal inflammation in driving melanoma stemness and chemoresistance, and advance the identification of effective treatment options currently lacking for patients with MM.

## Teaser

Mucosal inflammation induces melanoma plasticity with oncogenic addiction.

## INTRODUCTION

Melanoma represents a malignancy arising from melanocytes, the pigment-producing cells distributed across various tissues such as the skin, mucosa, and ocular structures (*1*). Although the incidence of melanoma continues to increase, the mortality rate has decreased over the past decade, owing to the breakthrough of checkpoint immunotherapy and BRAF-targeted therapies, especially in patients with advanced and high-risk stage III melanoma (*2*). However, notable responses to these treatments have been primarily observed in cutaneous melanoma. In contrast, noncutaneous melanoma subtypes, such as acral melanoma (melanoma on the hands and feet) (*3*) and mucosal melanoma (MM) (melanoma in the oral, nasal, gastrointestinal, and urogenital tracts) (*4*, *5*), have demonstrated significantly lower response rates to these therapies.

MM represents only 1% of melanomas in Caucasians but accounts for 22 to 25% of all melanomas in non-Caucasians, particularly in Asians (*4*). The oral and nasal mucosa are the most frequently affected anatomical areas, accounting for 55% of all MM cases (*6*–*8*). MM exhibits a markedly worse prognosis in comparison to cutaneous melanoma. Specifically, patients with MM face a grim 5-year overall survival (OS) rate of 14% after the initial diagnosis (*4*, *9*, *10*), in stark contrast to cutaneous melanoma with a 5-year survival rate exceeding 90% (*11*). This significant difference is driven by multiple factors, including the high rate of metastasis at diagnosis in patients with MM largely due to delayed detection in anatomically hidden sites (23 to 54% in MM versus 5 to 9% in cutaneous melanoma) (*12*, *13*), the absence of standardized staging protocols for MMs (*4*), and the limited response to various therapeutic modalities, including chemotherapy, targeted therapy, and immune checkpoint–based therapy (*14*–*16*). The precise contribution of cancer cell–intrinsic and microenvironmental factors to treatment resistance and poor prognosis in MM remains unclear.

In contrast to cutaneous melanoma, which is characterized by known hotspot driver mutations facilitating well-defined molecular subtyping, MM presents a notably dispersed genomic landscape, exhibiting a lower mutation burden dominated by large-scale structural variants that are not readily targetable (*17*, *18*). In addition, MM arises within a unique mucosal microenvironment that differs from the skin. Specifically, the mucosa serves as an actively inflamed soft tissue designed to defend against abrasion and infection (*19*). Taking oral mucosa as an example, in response to pathogens, pro-inflammatory macrophages and other phagocytic cells in the mucosa functionally engage in engulfing bacteria or viruses as well as induction of adaptive immune response. Following pathogen clearance, these phagocytic cells transition from pro- to anti-inflammatory phenotypes, thereby mitigating inflammation and restoring tissue integrity (*20*). However, in certain instances, even after pathogen removal, the inflammatory cascade may fail to deactivate, leading to uncontrolled and chronic mucosal inflammation observed in conditions such as periodontitis, a prevalent inflammatory disorder affecting the oral cavity (*21*, *22*). Chronic inflammation is also implicated in MMs and other mucosal-originated cancers, often referred to as a “wound that does not heal” (*23*). Nevertheless, the precise mechanisms underlying the interactions between mucosal inflammation–associated microenvironment and cancer cells, and their contribution to the exacerbation of MM progression, remain largely unknown.

In this study, we conducted an extensive analysis of single-cell transcriptomic profiles obtained from tumors of nine patients with MM. This was complemented by bulk mRNA profiles of tumors from an additional 45 patients with MM, followed by experimental validations using cells derived from patients with MM. Our primary objective was to systematically characterize MM subtypes, unraveling the nuanced pigment heterogeneity and plasticity inherent in cancer cells and their surrounding mucosal inflammatory microenvironment. Ultimately, our aim is to find targeted treatment modalities tailored specifically for MM.

## RESULTS

### Diminished pigmentation as an indicator of unfavorable prognosis in MM

**Fig. 1.**
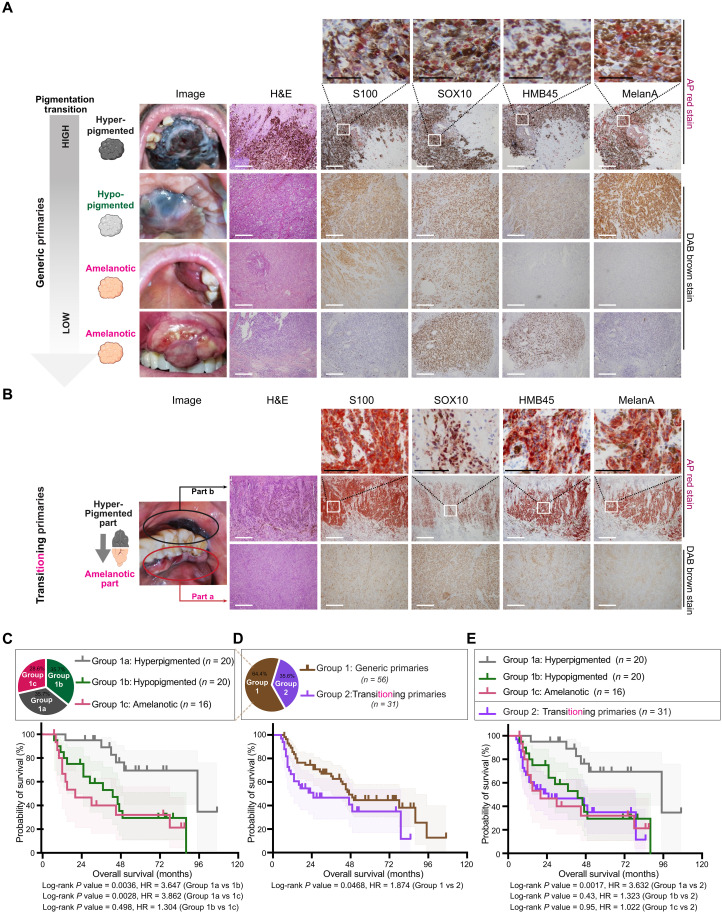
Decreased pigmentation as an indicator of unfavorable prognosis in MM. (**A** and **B**) H&E staining and IHC staining of MM tissues exhibiting varied pigment levels. (A) Generic primaries displaying a spectrum of pigment levels between individuals, from hyperpigmented to hypopigmented and amelanotic, accompanied by the expression of pigment-associated genes. (B) Transitioning primaries display varied pigment levels within individuals. Within the same patient, tumors with different pigment levels were independently isolated and pathologically evaluated. Representative H&E and IHC staining of patients with transitioning primaries. Scale bars, 100 μm (black dash) and 100 μm (white dash). AP red staining for hyperpigmented tissues; DAB brown staining for hypopigmented and amelanotic tissues. (**C**) Kaplan-Meier survival curve (log-rank test) comparing the OS among patients with hyperpigmented, hypopigmented, and amelanotic tumors in patients with generic primaries. Pigment scores between 31 and 100 define the hyperpigmented group, scores between 5 and 30 define the hypopigmented group, and scores below 5 define the amelanotic group. “*n*” represents the number of patients. Pie chart represents the ratio of patients with specific features as color-coded. (**D**) Kaplan-Meier curve (log-rank test) comparing the OS between patients with generic primaries and those with transitioning primaries. “*n*” represents the number of patients. Pie chart represents the ratio of patients with specific features as color-coded. (**E**) Kaplan-Meier survival curve (log-rank test) comparing the OS among patients with generic primaries (hyperpigmented, hypopigmented, and amelanotic tumors) and in patients with transitioning primaries. “*n*” represents the number of patients.

Our clinical observations revealed considerable variation in tumor colors, spanning from black, brown, and even to pink, indicating of pigment fluctuation in MM (Fig. 1A). Given that the visual observation of tumor darkness can be influenced by both pigments and the infiltration of blood vessels or other microenvironmental cells, a cost-effective pathological evaluation of pigment levels in MM is needed. As mucosa lacks hair follicles, the organ that is enriched with melanocytes and pigment particles, the brown coloring observed in the hematoxylin and eosin (H&E) staining image can faithfully represent melanin level as it is mainly synthesized by melanoma cells (*24*–*26*). Therefore, we used H&E stain and based on the extent of brown coloring to assess the pigment level (table S1). Tumors were categorized into three distinct subgroups based on the pigment score: (i) hyperpigmented tumors, characterized by universal and heavy pigmentation; (ii) hypopigmented tumors, demonstrating low pigmentation or presenting with a mosaic-like distribution of pigment within the tumors; and (iii) amelanotic tumors, characterized by a minimal or absent display of pigment. To our surprise, more than 60% of MM cases were identified in the hypopigmented or amelanotic groups, a prevalence far higher than that reported in cutaneous melanoma (~2%) (Fig. 1C, pie chart) (*24*–*27*). Examination of established melanoma markers revealed comparable levels of SOX10 staining across MM cases with varying pigment levels, whereas HMB45 and MelanA exhibited positive correlations with the tumor pigment score (fig. S1, A to C). Prognostic analysis showed that patients with hypopigmented and amelanotic tumors faced a poorer prognosis compared to their hyperpigmented counterparts (Fig. 1C). This result indicates that the loss of pigmentation correlates with a worse prognosis in MM. Given that the loss of pigmentation typically implies weakened lineage identity and increased cell dedifferentiation in cutaneous melanoma (*28*–*30*), we posit that a similar dedifferentiation event may occur pervasively in those MMs with decrease or loss of pigment (Fig. 1C). However, additional evidence is needed to comprehensively elucidate this observation.

Unexpectedly, we unveiled a noteworthy proportion of MM primaries exhibiting two or more isolated tumors with pronounced differences in pigment levels within the same patients (Fig. 1B). These cases were categorized as “transitioning primaries,” representing a snapshot of tumors that may actively undergoing transition from hyperpigmented to hypopigmented states. These patients with transitioning primaries have documented a medical history involving the initial observation of hyperpigmented hyperplasia, followed by the identification of another lesion with lower or nonpigmented features. Patients with “transitioning primaries” faced a significantly unfavorable prognosis, akin to those patients with hypopigmented or amelanotic primaries (Fig. 1, D and E). In addition, patients with higher American Joint Committee on Cancer (AJCC) staging tended to have higher portion of patients with transitioning primaries (fig. S1, D and E). The observed dynamic transition in pigment levels within the same patients may indicate an enhanced degree of phenotypic plasticity, a recognized hallmark of cancer associated with an unfavorable prognosis (*31*).

### Reduction of pigmentation coincides with increased neural crest stemness in MM

**Fig. 2.**
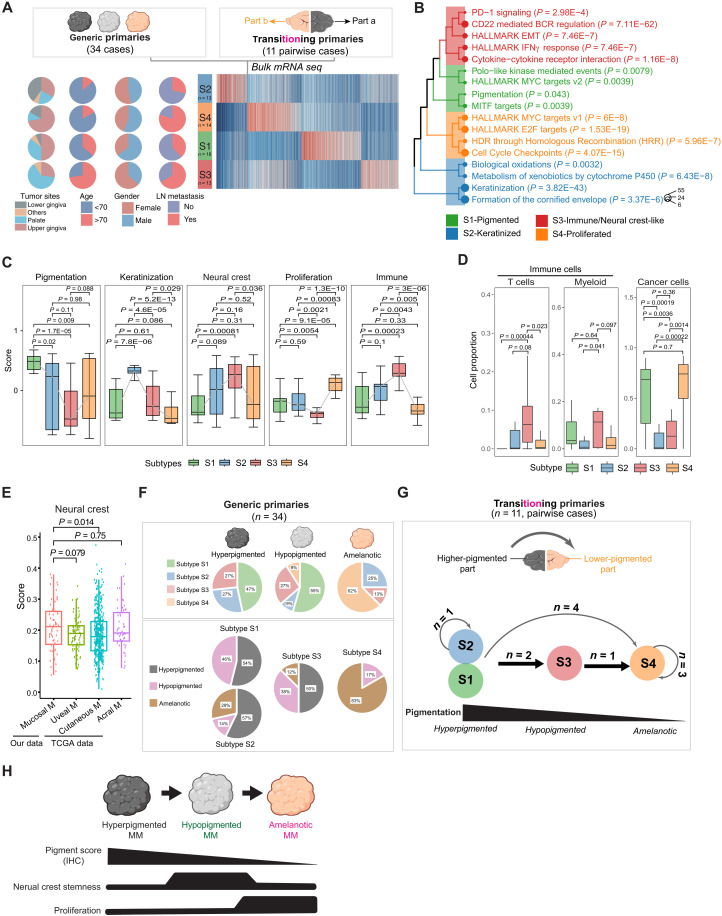
Reduction of pigmentation coincides with increased neural crest stemness in MM. (**A**) Unsupervised clustering of 45 MM samples using the top expressed genes, displaying four clusters labeled as S1 to S4. Samples include 45 cases with generic primaries and 11 pairwise cases with transitioning primaries. Pie charts on the left indicate clinical features of patients with MM, including tumor sites, age, gender, and lymph node (LN) metastasis. (**B**) Enriched GO terms for the four MM subtypes. Each color represents an indicated subtype, and the circle size indicates the number of enriched genes. (**C**) Enrichment score analysis of the four MM subtypes based on bulk mRNA-seq data. *P* values are from two-tailed *t* tests. Box and whisker plots represent mean, 10th and 90th percentiles, and minimum and maximum values of all correlated scores. (**D**) Predicted proportion of cancer cells and immune cells in each MM subtype by analyzing bulk mRNA-seq data using CIBERSORT. *P* values are from two-tailed *t* tests. (**E**) GSEA comparing the neural crest score across melanoma types based on bulk mRNA-seq data. Cutaneous M, cutaneous melanoma; uveal M, uveal melanoma; acral M, acral melanoma. (**F**) Pie chart depicting the composition of different MM subtypes within hyperpigmented, hypopigmented, and amelanotic samples (*n* = 34). (**G**) Schematic of the subtype distribution of higher-pigmented tumor parts and lower-pigmented tumor parts within the same patients with transitioning primaries. “*n*” represents the number of patients. The direction of the arrow represents higher-pigmented to lower-pigmented tumor part in the same patient with transitioning primaries (*n* = 11). (**H**) Schematic of mRNA expression programs during MM depigmentation. The scRNA-seq data for uveal melanoma (GSE139829) and cutaneous/acral melanoma (GSE215120) were obtained from the GEO NCBI database.

To elucidate the molecular mechanisms underlying the observed pigment heterogeneity in MM, we conducted bulk mRNA sequencing (mRNA-seq) analysis on a cohort of 45 patients with MM. This cohort included 34 patients with “generic primaries” and 11 patients with “transitioning primaries.” Notably, within the subset of 11 patients with transitioning primaries, the higher-pigmented and lower-pigmented tumor regions were independently sequenced (Fig. 2A, top). This approach enabled exploring the changes of gene expression programs during the transition from hyperpigmented to hypopigmented tumors.

Through unbiased clustering analysis of MM bulk mRNA data, we identified four distinct molecular subtypes of melanoma characterized by unique gene expression features (Fig. 2A). Gene ontology (GO) analysis (Fig. 2B and table S2) and single-sample gene set enrichment Analysis (ssGSEA) (Fig. 2C and table S3) were used to define these subtypes. Specifically, subtype S1 exhibited elevated Melanocyte Inducing Transcription Factor (MITF) activity and pigmentation gene signature, indicating a prevalence of pigmented melanoma cells within the tumor. Subtype S2 retained the pigmentation signature and exhibited high keratinization feature, suggestive of keratinization within the tumor region. Subtype S3 showed an elevated immune infiltration–related gene signature, suggesting a transition in cancer cell states and immune cell infiltration. Last, subtype S4 demonstrated high E2F target and cell cycle checkpoint features, indicating the enrichment of proliferating cells (Fig. 2, B and C). To validate these molecular subtypes, immunohistochemical (IHC) staining was performed on tumors from each subtype using subtype-specific markers: HMB45 for S1, KRT14 for S2, CD45 for S3, and Ki67 for S4 (fig. S2A). The staining results corroborated the features identified through the bulk mRNA-seq data, confirming the distinct biological characteristics of each subtype.

The dedifferentiation of cutaneous melanoma cells toward a neural crest stem cell (NCSC)–like state under therapeutic pressures, resulting in pigment loss, drug resistance, and poor prognosis, is a well-established feature in cutaneous melanoma (*28*, *29*, *32*, *33*). Accordingly, we used ssGSEA to evaluate the NCSC score by comparing mRNA expression programs in MM as well as other melanoma subtypes using a published neural crest gene list (table S4) (*34*). MM demonstrated the highest average neural crest score compared to other melanoma subtypes, including acral melanoma and skin cutaneous melanoma (SKCM), although the difference did not reach statistical significance (Fig. 2E). Within the MM subtypes, the S3 subtype displayed the highest neural crest score (Fig. 2C). Furthermore, we identified a positive correlation between the neural crest signature and the immune infiltration signature among the four subtypes (Fig. 2C and fig. S2B), specified by the myeloid cell and T cell infiltration as predicted by the CIBERSORT algorithm (Fig. 2D and fig. S2C). The above data indicate that myeloid cell or T cell infiltration might contribute to the enrichment of cancer cells at neural crest state in MM.

Because Tsoi *et al.* proposed an alternative subtyping strategy for cutaneous melanoma by categorizing cells into four states—C1 (differentiated), C2 (neural crest–like), C3 (transitory), and C4 (undifferentiated) (*28*)—we applied their signature gene sets to analyze bulk RNA sequencing (RNA-seq) data from MM cells. Our analysis revealed that MM S1 and S2 subtypes in MM exhibit the highest C1-melanocytic scores, aligning with melanocyte differentiation characteristics. The S3 subtype in MM showed a hybrid profile, incorporating traits from C2 (neural crest–like), C3 (transitory), and C4 (undifferentiated) states (fig. S2D). The S4 subtype in MM did not match any of the C1 to C4 subtypes described in Tsoi’s study. This indicates that, although MM shares some features with cutaneous melanoma, particularly in certain subtypes, it also displays distinct characteristics, underscoring the complexity and uniqueness of this melanoma subtype.

On the basis of average scores, transitioning primaries exhibited a higher neural crest signature—irrespective of whether the region was more or less pigmented—compared to generic primaries, except for the neural crest–high S3 cluster (fig. S2E). This observation suggests that a heightened neural crest signature may correlate with an increased tendency for pigment plasticity, subsequently leading to heterogeneous pigmentation levels within the transitioning primaries. Such plasticity might contribute to the adverse prognosis observed in this cohort, as depicted in Fig. 1D.

To evaluate the correlation between observed pigmentation levels and molecular subtyping, we analyzed the composition of the four subtypes within those generic primaries characterized by different pigment levels. Hyperpigmented melanomas were enriched with subtypes S1 and S2. Hypopigmented melanomas were enriched with subtype S3, whereas amelanotic melanomas were significantly enriched with subtype S4 (Fig. 2F). These findings suggest that, distinct from hyperpigmented melanomas, which tend to exhibit a strong pigmentation signature, hypopigmented melanomas tend to display a neural crest and immune infiltration signature, and amelanotic melanomas tend to manifest a proliferation signature.

To define the evolutionary trajectory of those molecular subtypes during the transition from hyperpigmented tumors to hypopigmented tumors, we took advantage of the 11 cases of transitioning primaries, each including two portions of tumors exhibiting different molecular subtypes, because these patients with transitioning primaries have documented a medical history involving the initial observation of hyperpigmented hyperplasia, followed by the identification of another lesion with lower or nonpigmented features. We posit a hypothesis that highly pigmented primary lesions, demonstrating the classical appearance of melanoma, may undergo a sequential transition toward less common hypopigmented primaries and eventually progress to amelanotic primaries. This potential evolution could be associated with a concurrent shift toward distinct molecular subtypes. Accordingly, by predefining the direction of transition from the more pigmented portion to the less pigmented portion within each primary tumor, we delineated an evolutionary trajectory progressing from subtype S1/S2 to subtype S3 and ultimately to subtype S4 (Fig. 2G). This trajectory also signifies a transition from hyperpigmented melanomas (characterized by a strong pigmentation signature) to hypopigmented melanomas (marked by a neural crest and immune infiltration signature) and finally to amelanotic melanomas (featuring a proliferation signature) (Fig. 2H).

### More enrichment of neural crest–like cancer cells in MM compared to other melanoma subtypes

**Fig. 3.**
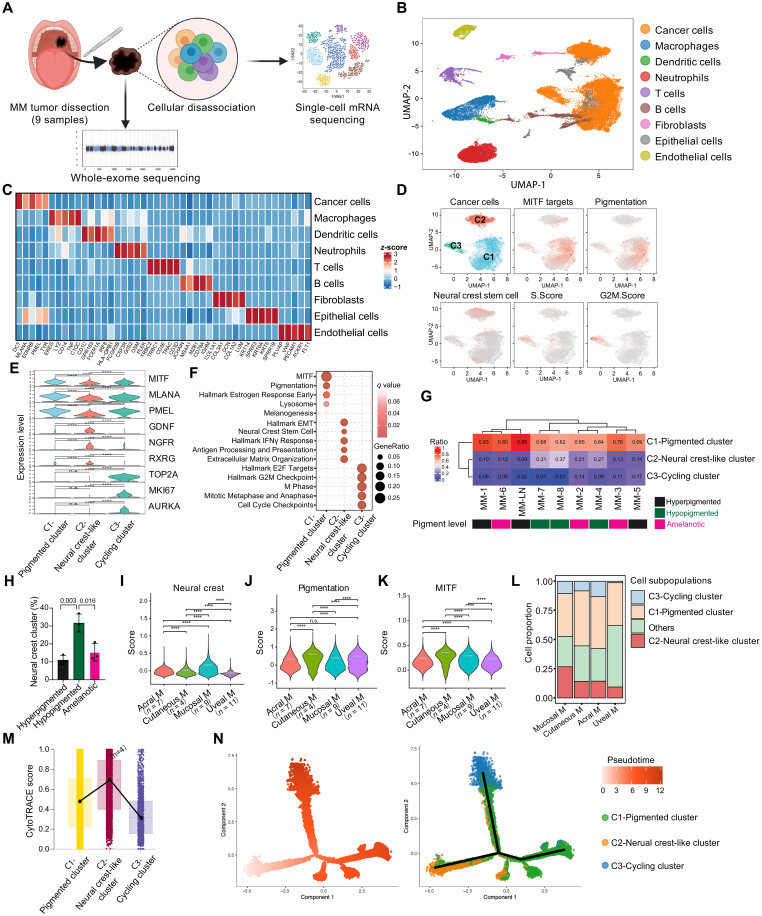
Increased neural crest–like cancer cells enrichment in MM compared to other melanoma subtypes. (**A**) Flowchart of scRNA-seq and WES of nine human MM samples. (**B**) UMAP plot of the single-cell mRNA-seq data from human MM samples. (**C**) Heatmap of the representative gene expression for each cell type. (**D**) UMAP plots showing the three different clusters of cancer cells with the corresponding gene set enrichment. Colors indicate subclusters. The color key from gray to red indicates low to high gene expression of correlated gene sets. (**E**) Violin plots for representative genes that are differentially expressed across the three cancer cell subclusters. (**F**) Enriched GO terms of signature genes in each cancer cell subcluster. Circle size reflects gene ratio of enriched genes within each GO term (bottom right). The color keys from white to red (top right) indicate the *P* value range. (**G**) Proportions of cancer cell subclusters in samples with different pigmentation levels (*n* = 9). (**H**) Bar plot comparing neural crest cluster proportions in hyperpigmented (*n* = 3), hypopigmented (*n* = 3), and amelanotic (*n* = 3) samples. (**I** to **K**) Neural crest (I), pigment (J), and MITF (K) scores compared across mucosal, cutaneous, uveal, and acral melanoma by integrative single-cell analysis. (**L**) Proportions of each cancer cell cluster across melanoma subtypes by integrative analysis of cancer cells from scRNA-seq data of each melanoma subtype. (**M**) CytoTRACE scoring of the three cancer cell subclusters. (**N**) Pseudotime analysis of the three MM cancer cell subclusters using Monocle 2. Cells are colored by subcluster and annotated to the right. Error bars represent SD of the mean. Cutaneous M, cutaneous melanoma; uveal M, uveal melanoma; acral M, acral melanoma. ****P < 0.0001

To further elucidate the correlation of cancer cell state with the enriched neural crest signature detected based on the MM bulk mRNA-seq data, we performed single-cell mRNA-seq of nine MM tumors, including eight tumors derived from oral mucosa and one tumor derived from lymph node metastasis (Fig. 3A and table S5). The goal was to characterize the diverse cancer cell states and investigate the intricate communication networks between the cancer cells and the specific microenvironmental cells present in the mucosa. In parallel, we performed comprehensive whole-exome sequencing (WES) to identify driver mutations in the MM samples. Consistent with the existing literature, MM exhibited lower tumor mutation burden than cutaneous melanoma (fig. S3A). Meanwhile, most identified pathogenic mutations were nontargetable, including *BRAF* non-V600E mutations observed in one of nine cases and *NRAS* mutations detected in three of nine cases (fig. S3B). In addition, akin to previous reports, frequent gene copy number changes were seen in MM exemplified as gene amplifications or deletion (*18*) (fig. S3C).

Using the single-cell mRNA-seq data, we characterized diverse cell types and their distinctive expression features within the MM samples, including cancer cells, fibroblasts, B cells, epithelial cells, neutrophils, T cells, and macrophages (Fig. 3, B and C, and fig. S3D). Intriguingly, the melanoma cells exhibited clear segregation into three distinct subclusters, denoted as C1, C2, and C3 (Fig. 3D and fig. S3E). Through scoring the signature of different functional programs, we uncovered distinct characteristics of each cancer cell subgroup (Fig. 3, D to F). Specifically, cluster C1 represented well-differentiated cancer cells enriched in pigmentation and melanogenesis-related genes. Cluster C2 demonstrated enrichment of neural crest signature genes, such as *NGFR*, *RXRG*, and *GDNF*, accompanied by the activation of neural crest stemness–associated transcription factors *JUNB*, *FOS*, and *ATF3* (fig. S3F). Cluster C3 displayed features of cell proliferation (Fig. 3, E and F, and table S6). All three cancer cell clusters were identified in each tumor sample, thereby eliminating the possibility of sample batch effects contributing to cancer cell clustering (Fig. 3G and fig. S3G). Meanwhile, despite the calculation being based solely on the nine single-cell RNA sequencing (scRNA-seq) samples, a higher proportion of neural crest–like cancer cells was observed in samples with hypopigmentation compared to hyperpigmented or amelanotic samples (Fig. 3, G and H), in line with the trends seen in the bulk mRNA-seq data presented in Fig. 2 (C to F).

By integrative analysis of single-cell mRNA-seq data of all melanoma cells from different melanoma subtypes, including cutaneous melanoma, acral melanoma, and uveal melanoma, we confirmed that cancer cells from MM, in general, showed the highest neural crest signature (Fig. 3I and fig. S3H), whereas the pigmentation-associated signatures were significantly lower than cutaneous melanoma cells (Fig. 3, J and K). Further subclustering of all cancer cells also showed that MM constituted the highest portion of neural crest–like melanoma cells compared to other melanoma subtypes (Fig. 3L). This finding corresponded to the highest neural crest score observed in MM compared to other melanoma subtypes based on bulk mRNA-seq data analysis, as depicted in Fig. 2E. The aforementioned data collectively demonstrate that the enrichment of neural crest–like melanoma cells strongly correlates with the widespread loss of pigmentation observed in MMs.

To analyze the potential transition trajectory between these three cancer cell states, we initially used CytoTRACE analysis, revealing that neural crest–like cancer cells were in a less differentiated state, whereas pigmented cancer cells and cycling cancer cells were in a more differentiated state (Fig. 3M). Time trajectory analysis unveiled that cancer cells could transition from the neural crest state to either the pigmented state or the cycling state (Fig. 3N). To validate this observed cancer cell state transition, we performed fluorescence-activated cell sorting (FACS) of neural crest–like cancer cells and nonneural crest state cells based on the NGFR expression, a well-accepted neural crest–like melanoma cells marker, from primary MM cells derived from the tumor. We observed a decrease in side scatter (SSC) score in NGFR-positive (NGFR+) cells (fig. S3I), indicating a decrease in pigmentation in those cells (*35*). Reverse transcription quantitative polymerase chain reaction (RT-qPCR) experiment confirmed the increased expression neural crest markers and decreased expression pigment markers in those sorted NGFR+ cells (fig. S3K). Subsequently, these sorted cells were cultured for 2 weeks and harvested for further analysis. Intriguingly, we observed a significant decrease in the proportion of NGFR+ cells within the pure NGFR+ cell population (fig. S3J, middle). This observation is consistent with previous findings in cutaneous melanoma (SKCM), where sorted NGFR+ melanoma cells gradually decrease NGFR expression over time (*36*, *37*). Meanwhile, an increase in NGFR+ cells was observed in the pure NGFR-negative (NGFR−) cell population (fig. S3J, middle). These findings underscore the dynamic potential of cancer cells to undergo bidirectional transitions between the neural crest–like state and the nonneural crest state in MM. Intriguingly, although expression of NGFR was decreased during the transition from NGFR+ to NGFR− cells, there was no significant elevation in the SSC score, indicating the maintenance of a low pigment state (fig. S3J, right). However, the NGFR− cells exhibited heightened proliferation signatures, as demonstrated by increased expression levels of *KI67* and *AURKB* (fig. S3L). The above data suggest that the enriched neural crest–like cancer cells in MM in orchestrating the transition to cycling cancer cells, concomitant with widespread pigment loss and unfavorable prognosis.

### TNF-α, expressed by macrophages, induces the neural crest–like state in MM cancer cells

**Fig. 4.**
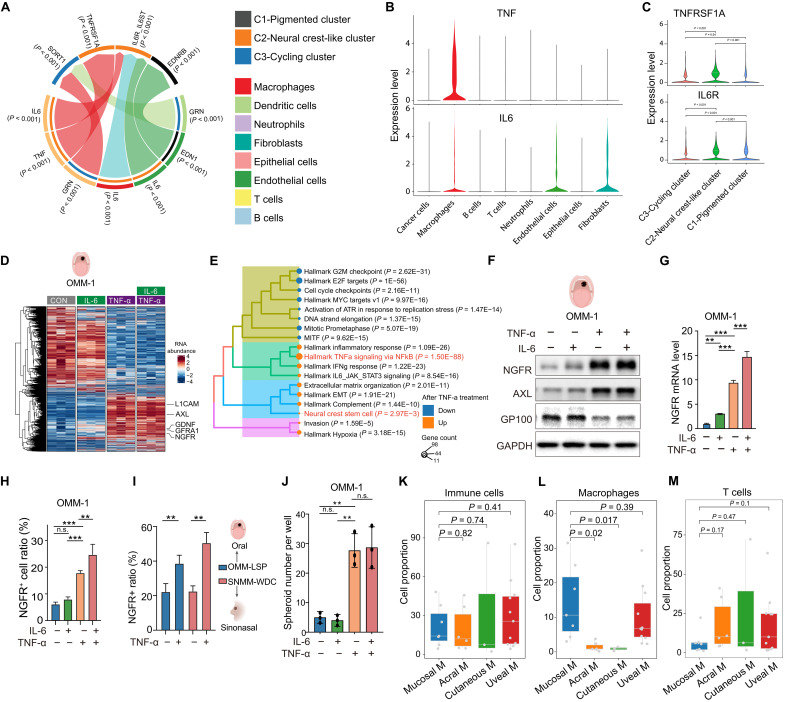
TNF-α, secreted by macrophages, induces the neural crest–like state in cancer cells of MM. (**A**) Cell-cell interaction analysis between three cancer cell clusters and microenvironmental cells. Populations are color coded (right), with arrows indicating ligand-receptor interactions. (**B**) Violin plots for *TNF* and *IL6* expression across all cell clusters in MM. (**C**) Violin plots for *TNFRSF1A* and *IL6R* expression across three cancer cell clusters. (**D**) Heatmap of gene expression changes in OMM-1 cells after 72 hours of TNF-α treatment (bulk RNA-seq; *n* = 3). Blue-red gradient shows low-high expression. (**E**) Enriched GO terms of DEGs between PBS-treated and TNF-α–treated OMM-1 cells. (**F**) Western blot detecting neural crest and pigment signature genes in the OMM-1 MM cell line untreated or treated with TNF-α, IL-6, or both for 72 hours. (**G**) RT-qPCR analysis of NGFR mRNA in OMM-1 cells untreated or treated with TNF-α, IL-6, or both for 72 hours (*n* = 3). (**H**) FACS analysis of NGFR+ cell proportions in OMM-1 cells untreated or treated with TNF-α, IL-6, or TNF-α/IL-6 combination for 72 hours (*n* = 3). (**I**) FACS analysis of NGFR+ cell proportions in OMM_LSP cells (oral MM cells) and SNMM_WDC cells (sinonasal MM cells) untreated or treated with TNF-α for 72 hours (*n* = 3). (**J**) Bar graph showing the number of spheroid formed in OMM-1 cells untreated or treated with TNF-α, IL-6, or both for 7 days (n = 3). (**K** to **M**) Bar graphs comparing proportions of immune cells (K), macrophages (L), and T cells (M) in MM versus acral, cutaneous, and uveal melanoma. Error bars represent SD of the mean. Statistical significance was determined using Student’s *t* test (*P < 0.05; **P < 0.01; ***P < 0.001; n.s., not significant).

To explore the microenvironmental factors contributing to the pronounced enrichment of neural crest–like cancer cells in MM, we conducted analysis of cell-cell communication between melanoma cells and the microenvironmental cells using scRNA-seq data of MM. Robust communication was observed between neural crest cancer cells and macrophages, fibroblasts, and endothelial cells, primarily mediated by the expression of pro-inflammatory cytokines tumor necrosis factor–α (TNF-α) and interleukin-6 (IL-6) (Fig. 4A). Among the various cell-cell communications observed, macrophages exhibited the highest level of communication with neural crest–like cancer cells (Fig. 4A). Macrophages displayed the highest expression of the *TNF* mRNA level compared to any other microenvironmental cells (Fig. 4B). Concurrently, neural crest–like cancer cells exhibited elevated expression levels of the IL-6 receptor (*IL6R*) and TNF-α receptor (*TNFRSF1A*) compared to nonneural crest–like cancer cells (Fig. 4C). Analysis of scRNA-seq–based cell-cell interactions between macrophages and cancer cells across Oral Mucosal Melanoma (OMM), Acral Melanoma (AM), and SKCM further revealed that MM harbored the strongest TNF-TNFRSF1A and IL-6–IL-6R interactions, suggesting a distinct microenvironmental cytokine landscape in this subtype (fig. S4B). Consistently, macrophages in MM expressed higher TNF than those in AM or SKCM (fig. S4A), underscoring the enrichment of TNF and IL-6 signaling in the mucosal context. Considering previous reports indicating that TNF-α induces the neural crest state in cutaneous melanoma (*38*, *39*), we explored whether similar outcomes could be observed in MM. Notably, treatment of the patient-derived MM line OMM-1 with TNF-α, but not IL-6, significantly enriched the neural crest signature based on bulk mRNA-seq data analysis (Fig. 4, D and E, and fig. S4C). TNF-α treatment also up-regulated the expression of *NGFR* and *AXL*, neural crest markers, at both mRNA and protein levels in OMM-1 and two other MM lines derived from oral and nasal mucosa (Fig. 4, F to I, and fig. S4D). Although IL-6 treatment alone had only a negligible effect on NGFR expression in OMM-1 cells, a modest additive increase was observed when combined with TNF-α, likely reflecting partial convergence on downstream pathways regulating NGFR. Rather than exerting its classical role in promoting cytotoxicity or growth inhibition in certain tumor contexts (*40*), TNF-α instead enhanced tumorsphere formation in MM cells, supporting its role in driving a neural crest–like stem cell state (Fig. 4J and fig. S4, E and F). To assess whether this phenomenon extends beyond MM, we examined TNF-α–induced NGFR expression in additional melanoma models, including two cutaneous melanoma lines (SKMEL-28 and A375) and two uveal melanoma lines (MUM2B and 92-1). RT-qPCR analysis revealed TNF-α–induced NGFR up-regulation only in SKMEL-28 cells but not in the other lines (fig. S4G). These findings indicate that the TNF-α–driven neural crest–like state in MM is not broadly conserved across melanoma subtypes.

In line with our in vitro findings, we performed intratumoral injections of TNF-α into OMM-1 MM xenografts. TNF-α–treated tumors exhibited enhanced TNF-α staining, confirming successful delivery (fig. S4H). As expected, injection of TNF-α into tumors also increased the NGFR level, decreased the MelanA level, and increased macrophage infiltration as illustrated by F4/80 staining (fig. S4, I and J). By contrast, the spatial distribution of stromal cells remained largely unchanged (fig. S4K). In addition, MM owned the highest macrophage infiltration among all melanoma subtypes, explaining why it has the highest portion of neural crest–like cancer cells (Fig. 4, K and L). Besides macrophages, MM had the lowest infiltration of T cells, elucidating why patients with MM lack response to immune checkpoint inhibitors (Fig. 4M and fig. S4L).

### TNF-α–secreting COX2+ macrophages are induced by mucosal inflammation during melanoma progression

**Fig. 5.**
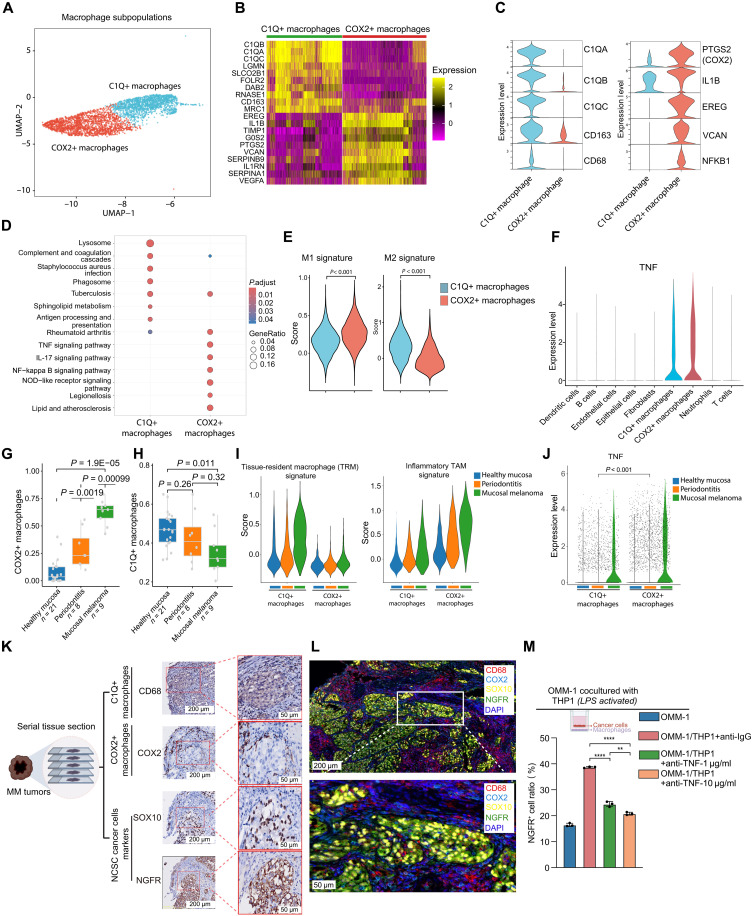
COX2+ macrophage subpopulation secretes the highest level of TNF-α and is induced by mucosal inflammation during melanoma progression. (**A**) UMAP plots illustrating two distinct subclusters of MM-associated macrophages named COX2+ macrophages and C1Q+ macrophages, represented by different colors. (**B**) Heatmap showing top DEGs between COX2+ and C1Q+ macrophages based on MM scRNA-seq data. (**C**) Violin plots displaying representative genes that exhibit differential expression across the two macrophage subclusters. (**D**) Enriched GO terms for DEGs between COX2+ and C1Q+ macrophages based on MM scRNA-seq data. (**E**) Enrichment score analysis of the M1 macrophage signature (*64*) and M2 macrophage signature (*64*) for COX2+ and C1Q+ macrophages based on MM scRNA-seq data. (**F**) Violin plots of TNF expression across all microenvironmental cells. (**G** and **H**) Bar graphs representing the proportions of COX2+ macrophages (G) and C1Q+ macrophages (H) among monocyte-derived cells in healthy mucosa, periodontitis, and MM. (**I**) GSEA of the TRM signature (*64*) (left) and inflammatory TAM signature (*64*) (right) for C1Q+ macrophages and COX2+ macrophages in healthy mucosa, periodontitis, and MM. TRM, tissue-resident macrophages; TAM, tumor-associated macrophages. (**J**) Violin plots displaying the *TNF* expression in C1Q+ macrophages and COX2+ macrophages across healthy mucosa, periodontitis, and MM. (**K**) IHC staining of CD68 (C1Q+ macrophage marker), COX2 (COX2+ macrophage marker), SOX10 (melanoma marker), and NGFR (neural crest–like melanoma marker) in MM tumors using serial tissue sections. (**L**) Multiplex immunofluorescence staining of CD68, COX2, SOX10, NGFR, and DAPI with the indicated fluorescence channels. (**M**) FACS analysis showing the proportion of NGFR+ OMM-1 melanoma cells after coculture with M1-polarized THP-1 macrophages, with or without TNF-α–neutralizing antibody treatment. Error bars represent SD of the mean.

To further elucidate the specific features of *TNF*-expressing macrophages within MM, we conducted a higher-resolution clustering of macrophages based on single-cell mRNA-seq data. Two distinct subpopulations were identified: C1Q-positive (C1Q+) macrophages and COX2-positive (COX2+) macrophages (Fig. 5, A and B). Differential expression gene analysis of these subpopulations revealed that C1Q+ macrophages are primarily involved in phagocytosis (*C1QA*, *MSR1*, *FCGR1A*, *FCGR2A*, *RAB5*, etc.) and antigen presentation (*HLA-DPB1*, *HLA-DPA1*, *HLA-DMB*, *B2M*, etc.), whereas COX2+ macrophages were enriched with genes in pro-inflammatory pathways, including the TNF-α/nuclear factor κB (NF-κB) pathway (*PTGS2*, *IL1B*, *CCL20*, *TNFRSF1B*, *NFKB1*, etc.) and IL-17 pathway activation (*CXCL2/3/5/8*, *CCL20*, *IL6*, *S100A8*, etc.) (Fig. 5, C and D). Meanwhile, C1Q+ macrophages exhibited a higher anti-inflammatory M2 macrophage-associated gene signature, whereas COX2+ macrophages displayed a higher pro-inflammatory M1 macrophage gene signature (Fig. 5E). Moreover, TNF-α is predominantly expressed in COX2+ macrophages compared to C1Q+ macrophages (Fig. 5F and fig. S5A), indicating the major contribution of COX2+ macrophages in driving the transition of MM cells to a neural crest state via TNF-α.

To elucidate whether the COX2+ macrophages were resident in normal mucosa, we conducted a comparative analysis of macrophage subpopulations between normal mucosa and MM through the integration of scRNA-seq data from both tissues (fig. S5B). We found a sharp increase in myeloid cells in MM than in normal mucosa (fig. S5C). Meanwhile, the COX2+ macrophages were scarcely observed in normal mucosa but were prominently enriched in MM (Fig. 5G). Conversely, C1Q+ macrophages constituted the primary macrophage subpopulation in normal mucosa but exhibited a significant decrease in abundance in MM (Fig. 5H). These findings indicate that C1Q+ macrophages may serve as tissue-resident macrophages in the mucosa, whereas COX2+ macrophages are elicited in response to MM initiation. A significantly higher tissue-resident macrophage signature was identified in C1Q+ macrophages compared to COX2+ macrophages (Fig. 5I, left).

Given the enrichment in pro-inflammatory gene expression in COX2+ macrophages as shown in Fig. 5D, we postulate that these COX2+ macrophages are induced in response to local mucosal inflammation during tumorigenesis. To investigate this hypothesis, we incorporated single-cell sequencing data from a cohort with periodontitis, a common disease characterized by chronic inflammation in the oral mucosa, and analyzed all monocyte-derived cells. Intriguingly, akin to MM, an increased proportion of COX2+ macrophages and a decreased proportion of C1Q+ macrophages were also observed in periodontitis compared to healthy mucosa (Fig. 5, G and H). Furthermore, we identified significantly higher inflammatory tumor-associated macrophage (TAM) signatures in COX2+ macrophages compared to C1Q+ macrophages (Fig. 5I, right). The inflammation index of COX2+ macrophages exhibited a gradient increase from normal mucosa to periodontitis and further to MM (Fig. 5I, right), exemplified by the highest mRNA level of *TNF* in COX2+ macrophages within MM (Fig. 5J). IHC staining using serial tissue sections of MM, as well as multicolor immunofluorescence staining followed by data quantifications, provided evidence that NGFR+SOX10+ neural crest–like MM cells are closely surrounded by COX2+ macrophages but are situated farther away from C1Q+ macrophages, as indicated by COX2 or CD68 staining for each subtype (Fig. 5, K and L, and fig. S5, D to G). Multiplex immunofluorescence further confirmed that TNF-α was specifically expressed in the vicinity of COX2+ macrophages (fig. S5H). To functionally mimic their induction, we differentiated THP-1 cells and peripheral blood mononuclear cell (PBMC)–derived monocytes into pro-inflammatory M1 macrophages using lipopolysaccharide (LPS), which led to a robust increase in the coexpression of COX2 and TNF-α (fig. S5, I and L). Coculture experiments further confirmed that the activation of pro-inflammatory M1 macrophages could induce MM cells toward the NGFR+ neural crest–like state (fig. S5, M and N). Accordingly, blocking TNF-α binding to cancer cells with a TNF-α–neutralizing antibody effectively reversed macrophage-induced neural crest stemness, as indicated by the reduction in NGFR expression (Fig. 5M and fig. S5, O and P). In summary, pigment loss in MM is associated with a cellular transition from a pigmented state to a neural crest–like state, driven by infiltrating COX2+ macrophages and their secretion of TNF-α (fig. S5Q).

### Neural crest–like cancer cells are addicted to HER2 and HER3 in MM

**Fig. 6.**
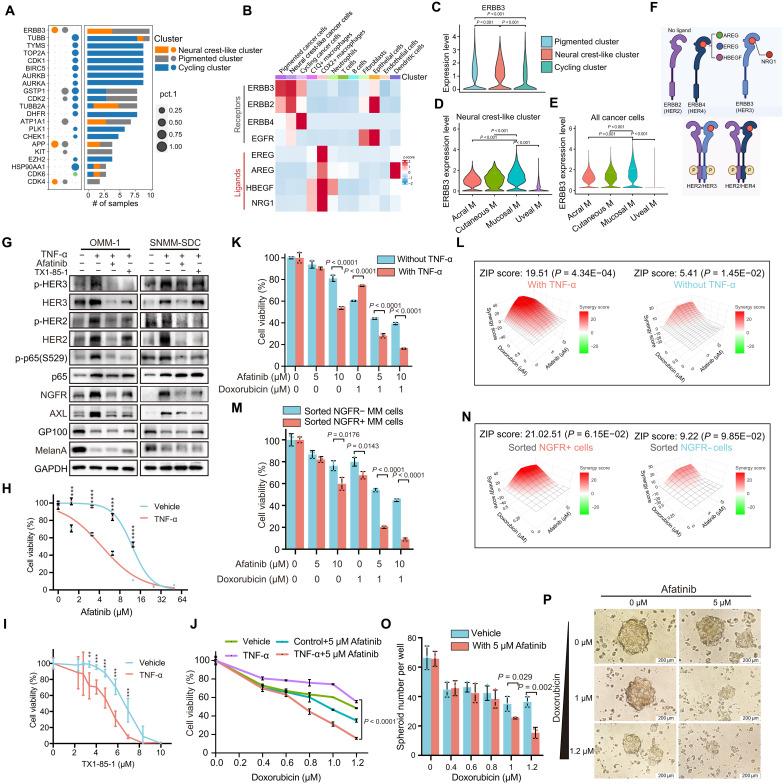
Neural crest–like cancer cells are addicted to HER2 and HER3 in MM. (**A**) Drug target prediction based on highly expressed genes in three mucosal melanoma cancer cell clusters by scRNA-seq. Circle size indicates the expression level; bar graph shows the proportion and number of samples. Cell clusters are color coded. (**B**) Heatmap of HER family member and ligand expression across clusters. (**C**) Violin plots of HER3 expression across three cancer cell clusters. (**D** and **E**) Violin plots comparing HER3 expression in the neural crest cancer cell cluster (D) or all cancer cells (E) across melanoma subtypes. (**F**) Schematic of HER ligand-receptor interactions (top) and receptor dimerization (bottom). (**G**) Western blot of neural crest, pigment, NF-κB pathway, and HER2/HER3 pathway genes upon treatment with TNF-α, pan-HER inhibitors (afatinib and TX1-85-1), or combinations in OMM-1 and SNMM-SDC cells. Cells were treated with TNF-α (25 ng/ml, 72 hours) and then afatinib (5 μM) or TX1-85-1 (5 μM, 24 hours). (**H** and **I**) Dose-response curves showing increased afatinib (H) and TX1-85-1 (I) sensitivity in OMM-1 after TNF-α treatment (*n* = 3). (**J**) Cell viability of OMM-1 cells with doxorubicin upon cotreatment with TNF-α, afatinib, or combinations for 72 hours (*n* = 3). (**K** and **L**) Bar plot (K) and synergy score (L) of cell viability changes upon combined treatment of afatinib and doxorubicin, with or without TNF-α (*n* = 3). (**M** and **N**) Bar plot (M) and synergy score (N) of cell viability changes after treatment with afatinib, doxorubicin, or their combinations in FACS-sorted NGFR+ OMM-1 cells or FACS-sorted NGFR− OMM-1 cells (*n* = 3). (**O** and **P**) Bar graph (O) and images (P) showing spheroid formation in OMM-1 cells treated with afatinib, doxorubicin, or combination for 6 days (*n* = 3). Scale bars, 200 μm. Error bars represent SD of the mean. *P < 0.05; **P < 0.01; ***P < 0.001, unpaired *t* test; n.s., not significant.

The transition from a differentiated state to a neural crest–like state is acknowledged as a substantial contributor to resistance against targeted therapy and chemotherapy in cutaneous melanoma (*28*, *29*, *32*, *33*). Similarly, augmented resistance to doxorubicin, an anthracycline chemotherapy drug, was observed in the TNF-α–treated MM cell line compared to their nontreated counterparts as shown in Fig. 6 (K and J). Therefore, eradicating those drug-resistant neural crest–like cancer cells holds the potential for improved clinical outcomes in the management of MM. To identify drug targets tailored for neural crest–like MM cells, we analyzed their expression patterns based on the scRNA-seq data. This analysis led to the identification of *ERBB3* (Erb-B2 receptor tyrosine kinase 3, expressed the protein named HER3), a member of the HER family, as the top candidate for targeting neural crest–like cancer cells (Fig. 6A). HER3 functions by activating downstream signaling pathways through dimerization with other members of the HER family, including epidermal growth factor receptor (EGFR), ERBB2 (or HER2), and ERBB4 (or HER4). Moreover, both HER2 and HER3 exhibited higher expression levels in neural crest–like cancer cells compared to nonneural crest–like counterparts in MM (Fig. 6, B and C). Furthermore, those neural crest–like cancer cells within MM showed higher HER3 expression level than cells within cutaneous melanoma or other melanoma subtypes (Fig. 6, D and E). Given that the activation of HER family members relies on specific ligand binding (Fig. 6F), our subsequent analysis aimed to identify the cells expressing HER ligands using our scRNA-seq dataset. COX2+ macrophages, previously identified as crucial contributors in induction of neural crest–like cancer cells in MM, emerged as the principal source of HER ligand expression, including *AREG*, *EREG*, *HBEGF*, and *NRG1* (Fig. 6B). This observation suggests that COX2+ macrophages exhibit a versatile array of functions, including stimulating the transition of cancer cells to the neural crest–like state via TNF-α, as well as the induction of HER activation through their ligand secretion. Furthermore, in vitro experiments demonstrated that TNF-α could up-regulate HER2 and HER3 expression mainly by increasing their mRNA levels in MM cells (Fig. 6G and fig. S6A). Collectively, the presented data herein delineate a multifaceted activation of the HER signaling pathway in neural crest–like melanoma cells, orchestrated by the secretome of COX2+ macrophages.

To further substantiate whether neural crest cancer cells in MM rely on activated HER signaling to sustain their stemness and growth, we administered a panel of HER inhibitors to MM cells, including afatinib (EGFR/HER2/HER4 inhibitor), allitinib (EGFR/HER2/HER3 inhibitor), and TX1-85-1 (HER2/HER3 inhibitor) (*41*). These HER inhibitors demonstrated efficacy in reversing TNF-α–induced NGFR and AXL expression, inhibiting the downstream NF-κB signaling in both OMM-1 and SNMM-SDC cells (Fig. 6G and fig. S6B), as well as inhibiting TNF-α–induced tumorsphere formation in OMM-1 cells (fig. S6, D and E). Furthermore, cancer cells exhibited increased sensitivity to HER inhibitors upon transitioning to a neural crest–like state via TNF-α in vitro (Fig. 6, H and I, and fig. S6C). Because afatinib targets not only HER2/3 but also EGFR and other RTKs, we evaluated whether TNF-induced NCSC transition might depend on additional receptors. scRNA-seq analysis revealed relatively low expression of EGFR, MET, and AXL compared with HER3 across MM subpopulations (fig. S6F). Consistent with this, TNF treatment of OMM-1 cells up-regulated AXL, HER2, and HER3 but not EGFR or MET (fig. S6A), suggesting that EGFR and MET are unlikely to play a dominant role. To functionally dissect receptor involvement, we performed short hairpin RNA (shRNA)–mediated knockdown of HER2, HER3, AXL, EGFR, and MET under both phosphate-buffered saline (PBS) and TNF treatment. RT-qPCR confirmed efficient knockdown of each target (fig. S6G). Notably, TNF-induced NGFR expression was specifically abrogated by HER2 or HER3 knockdown but not by knockdown of AXL, EGFR, or MET (fig. S6H). Together, these results establish HER2 and HER3 as the predominant drivers of TNF-mediated NCSC state induction, supporting the specificity of our findings beyond pharmacologic inhibition with afatinib.

### Combining the HER family inhibitors with chemotherapy elicits synergistic elimination of neural crest–like cancer cells

Until now, clinical trials focusing on targeted therapy and immune checkpoint blockade have not obtained official approval specifically for MM, primarily due to a lack of satisfactory response therapy (*14*–*16*). Consequently, chemotherapy remains the predominant first-line treatment for patients with MM, albeit with limited efficacy. The urgent need for strategies to enhance efficacy of chemotherapy in patients with MM is evident. Most commonly used chemotherapies in cancer—including dacarbazine, temozolomide, and platinum-based agents—showed little activity in OMM-1 MM cells (fig. S6I), in line with the poor clinical responses seen in patients. Anthracyclines (doxorubicin, daunorubicin, and idarubicin) exhibited meaningful activity in vitro (fig. S6J), suggesting their potential benefit in MM.

Considering that melanoma cells in a neural crest state tend to exhibit increased resistance to chemotherapy, we hypothesize that TNF-α–induced neural crest–like cancer cells may confer resistance even to anthracyclines, which are otherwise active in MM, and eradicating neural crest–like cancer cells via HER inhibition may sensitize MM cells to anthracyclines. In line with this, greater cell-to-cell variability in response to doxorubicin was observed following TNF-α induction (Fig. 6K). Meanwhile, cotreatment with the HER family inhibitor afatinib and doxorubicin produced marked synergistic effects in TNF-α–treated MM cells (Fig. 6, K, L, and J, and fig. S6K). Similar synergistic effects were also noted with other chemotherapy drugs, including daunorubicin and idarubicin (fig. S6L). Consistent results were observed when comparing FACS-sorted NGFR+ MM cells (representing the neural crest–like state) with NGFR− cells (representing the nonneural crest state) (Fig. 6, M and N). Moreover, we observed a combined inhibitory effect on TNF-α–induced spheroid formation after cotreatment of afatinib and doxorubicin (Fig. 6, O and P).

### In vivo validation of a pan-HER inhibitor combined with chemotherapy in an MM PDX model

**Fig. 7.**
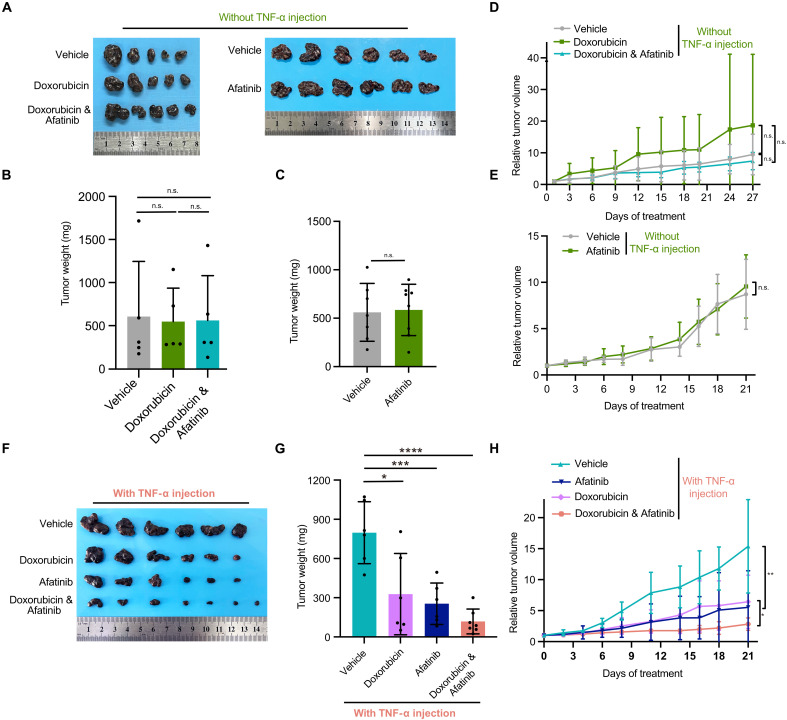
In vivo validation of a pan-HER inhibitor combined with chemotherapy in an MM PDX model. (**A** to **E**) Evaluation of tumor growth inhibition by treatment with afatinib, doxorubicin, or their combination in the OMM-1 PDX model without TNF-α injection into the tumors. Tumor images (A), tumor weight [(B) and (C)], and relative tumor volume [(D) and (E)] were assessed. (**F** to **H**) Evaluation of tumor growth inhibition by treatment with afatinib, doxorubicin, or their combination in the OMM-1 PDX model with TNF-α injection into the tumors. Tumor images (F), tumor weight (G), and relative tumor volume (H) were assessed. Error bars represent SD of the mean. *P < 0.05; **P < 0.01; ***P < 0.001, paired *t* test. n.s., indicates no significant difference (last timepoint of the relative tumor volume was used for *t* test evaluation).

To evaluate the in vivo effect of pan-HER inhibitor in MM, the OMM-1 MM patient-derived xenograft (PDX) model was established. Singular administration of afatinib or doxorubicin did not elicit significant tumor growth inhibition (Fig. 7, A to E, and fig. S7, A and B). However, subsequent TNF-α treatment sensitized tumors to afatinib, resulting in a synergistic effect observed in inhibiting tumor growth upon combination therapy with afatinib and doxorubicin (Fig. 7, F to H). The drug combination was well tolerated by mice, as evidenced by the absence of weight loss posttreatment (fig. S7C). Considering the established safety profile of afatinib in other cancer indications with Food and Drug Administration (FDA) approval (*42*, *43*), these findings advocate for the expedited clinical validation of this synergistic therapeutic approach in the management of patients with MM.

## DISCUSSION

### MM is a rare and highly aggressive malignancy that exhibits significant heterogeneity

**Fig. 8.**
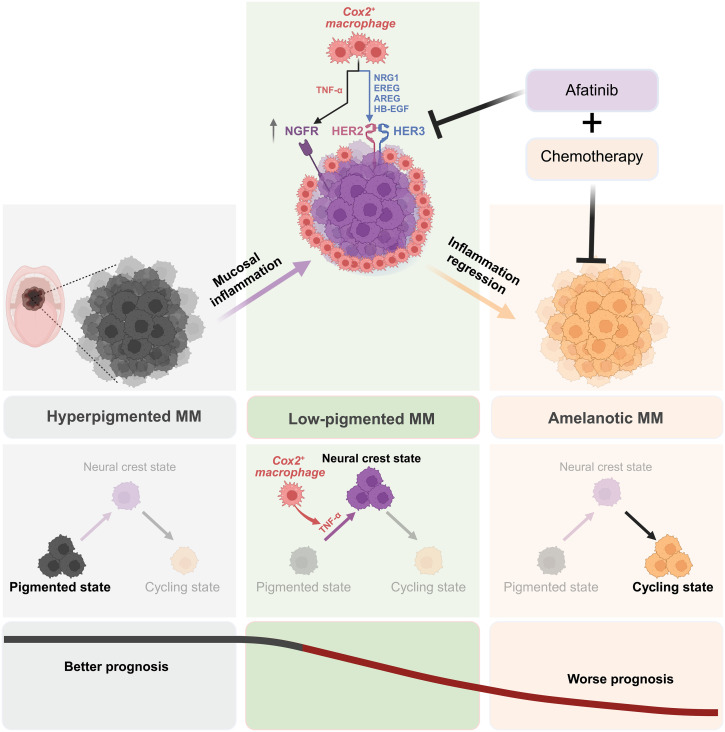
Model depicting tissue-specific inflammation inducing cell state plasticity with oncogenic dependency in MM. On the basis of the data presented here, we propose a model wherein mucosal inflammation–associated COX2+ macrophages promote the emergence of a more aggressive neural crest–like cancer cell state, characterized by the addiction to HER2 and HER3 oncogenic signaling, in MM.

In contrast to cutaneous melanoma, MM has presented notable challenges in terms of treatment efficacy, displaying limited responsiveness to chemotherapy and resulting in unfavorable prognoses. The scarcity of available clinical samples, cancer cell lines, and subtype-specific mouse models has further impeded comprehensive investigations into MM, encompassing molecular subtyping, characterization of the tumor microenvironment, and the identification of potential therapeutic targets. Our study commenced with an exploration of the distinctive tumor pigment heterogeneity observed in MM, specifically the prevalence of hypopigmented or amelanotic tumors. Notably, we observed substantial plasticity in pigment levels within primary tumors and throughout disease progression, aligning with a correlated worsened prognosis. Through the integration of bulk mRNA-seq and single-cell mRNA-seq analysis of patients with MM, we delineated four distinct subtypes based on their expression patterns. Crucially, our investigation uncovered a significantly elevated NCSC signature in MM compared to cutaneous melanoma, elucidating the heightened pigment plasticity observed in MM. Mechanistically, our findings unveiled an enrichment of COX2+ macrophages in MM, representing an M1-like macrophage subtype induced by mucosal inflammation. These COX2+ macrophages were found to induce the transition of MM cells into HER-addicted neural crest–like cancer cells and promote chemoresistance through the secretion of TNF-α and HER ligands. Targeting HER2/3 signaling demonstrated promising results in eradicating these neural crest–like cancer cells and reversing chemoresistance in MM (as summarized in Fig. 8). These insights provide a deeper understanding of MM pathogenesis and offer potential avenues for targeted therapeutic interventions, warranting further investigation and development.

### Correlation between bulk RNA-seq–based subtyping and scRNA-seq cancer cell states

In Fig. 2, we identified four molecular subtypes (S1 to S4) using bulk RNA-seq data from 56 MM samples. This classification reflects transcriptional programs arising from both cancer cells and tumor-microenvironment components. In contrast, scRNA-seq analysis of nine samples (Fig. 3) resolved three melanoma cancer cell states (C1 to C3). Because the bulk RNA-seq and scRNA-seq datasets were generated from independent patient cohorts, these classification systems cannot be directly matched at the sample level. Instead, their relationship is inferred conceptually by linking bulk subtype–defining transcriptional programs to dominant cancer cell states identified by scRNA-seq while accounting for microenvironmental contributions to bulk profiles. Subtypes S1 (pigmented) and S4 (cycling) contain a high proportion of cancer cells (>50%; Fig. 2D), making their bulk transcriptional signatures more reflective of cancer cell–intrinsic programs corresponding to the C1 pigmented and C3 cycling states, respectively. The S3 immune/neural crest–like subtype shows the highest neural crest–like (NCSC) score, consistent with enrichment of the C2 neural crest–like cancer cell population identified by scRNA-seq. The S2 keratinized subtype represents a distinct scenario in which the defining transcriptional signature is driven by keratinocyte enrichment rather than a melanoma cell–intrinsic state. Supporting this interpretation, IHC analysis shows strong KRT14 staining in S2 tumors, whereas HMB45 staining remains comparable to S1 tumors and higher than S3 and S4 tumors (fig. S2A), indicating the presence of pigmented melanoma cells alongside abundant keratinocytes. Together, these observations support a working model linking bulk and single-cell classifications in MM: S1/S2 tumors are associated with C1 pigmented cancer cells enrichment, S3 tumors are associated with C2 neural crest–like cancer cells enrichment, and S4 tumors are associated with C3 cycling cancer cells enrichment.

### Enrichment of neural crest–like melanoma cells beyond drug resistance

In melanoma cells, the adoption of a neural crest–like state has been correlated with cellular dedifferentiation and the hijacking of gene regulatory networks reminiscent of the neural crest–like embryonic structure from which melanocytes originate (*44*). The induction of neural crest–like melanoma cells has been extensively documented as a primary contributor to drug resistance in targeted therapies for cutaneous melanoma (*28*, *29*, *32*, *33*). However, the precise mechanisms by which tumor microenvironmental cells induce neural crest–like cancer cells remain incompletely understood. In this study, we have elucidated a significantly higher prevalence of neural crest–like cancer cells in MM compared to cutaneous melanoma of the skin and other melanoma subtypes. A substantial proportion of patients who contributed tumor samples for sequencing had not undergone any treatment, suggesting that the neural crest–like cancer cells associated with MM are not solely induced during the course of drug resistance. Our investigations have revealed that mucosa-associated macrophages play a pivotal role as the primary source for inducing neural crest–like MM cells through the secretion of TNF-α. These findings offer an illustrative example of how neural crest–like melanoma cells can be induced without the influence of treatment pressure. The identification of mucosa-associated macrophages as key contributors to this induction provides valuable insights into the complex interplay between the tumor microenvironment and melanoma cell behavior, shedding light on potential avenues for targeted therapeutic interventions.

### Neural crest–like melanoma cells in MM have unique features that can be leveraged for targeted intervention

The presence of neural crest–like cancer cells in MM has been linked to drug resistance, a phenomenon similarly observed in cutaneous melanoma therapy (*14*–*16*). Addressing the challenge of eradicating these resilient neural crest–like cancer cells remains a major concern in the melanoma field. Previous studies by Rambow and others have explored the efficacy of targeting retinoid X receptor (RXR) inhibitors, histone deacetylase (HDAC) inhibitors, and ferroptosis inducers to selectively inhibit neural crest–like melanoma cells (*28*, *29*, *32*, *33*). However, clinical evidence of efficacy is still needed to establish the feasibility of these approaches. In our study, we identified that HER2 and HER3 play pivotal roles in supporting the growth of neural crest–like cancer cells in MM. This dependency can be attributed, at least in part, to the unique microenvironment of MM, particularly the presence of COX2+ macrophages surrounding cancer cells, leading to the constant secretion of HER ligands. Notably, high expression of HER3 was also observed in neural crest–like melanoma cells across various cutaneous melanoma and melanoma subtypes (Fig. 6, D and E). Consequently, beyond overcoming chemoresistance in MM, HER inhibition also holds the potential for overcoming resistance to first-line therapies in cutaneous melanoma and other melanoma subtypes, such as BRAF/MEK inhibitors in BRAFV600E mutant cutaneous melanoma. However, the extension of our findings to encompass all melanoma subtypes necessitates further validation.

### Targeting inflammation-associated COX2+ macrophages shows promise as a therapeutic approach for the treatment of MM

Mucosal tissues have distinct mucosal immunity mechanisms actively defending against bacterial threats. Using oral mucosa as an example, an imbalance in this immunity can lead to inflammation, resulting in conditions such as periodontitis and potentially cancer. The relationship between infection, inflammation, and periodontitis has been extensively explored (*21*, *45*). However, the specific role of mucosal inflammation in contributing to the progression of MM remains elusive. Our investigation focuses on macrophages due to the notable communication observed between macrophages and neural crest–like cancer cells, facilitated by inflammatory factors TNF-α and IL-6. TNF-α, extensively studied, has been shown to induce the transition of cancer cells to the neural crest state, imparting resistance against targeted therapies and immune checkpoint blockade (*38*, *39*). This phenomenon has been validated in primary MM cells derived from both oral and nasal regions (Fig. 4I and fig. S4D). Given the enrichment of macrophages in normal mucosa, we reanalyzed scRNA-seq data of normal mucosa, periodontitis, and MM to investigate macrophage subtypes. We found a switch in macrophage subtypes in MM, transitioning from C1Q+ macrophages to COX2+ macrophages in response to mucosal inflammation observed in both periodontitis and MM. This finding is notable as it reveals the induction of COX2+ macrophages alongside mucosal-specific inflammation, playing a pivotal role in driving MM cells toward the neural crest–like state. This transition ultimately contributes to tumor pigment plasticity and drug resistance. Simultaneous targeting of COX2+ macrophages and melanoma cells emerges as a promising and specific therapeutic strategy for MM.

## MATERIALS AND METHODS

### Human subjects

The procedures for human subject research, tissue handling, and data analysis were performed as previously described (*46*). Samples of tumor were obtained from surgical specimens of patients undergoing resection at Shanghai Ninth People’s Hospital Affiliated to Shanghai Jiao Tong University School of Medicine after obtaining informed consent with a protocol reviewed and approved by the Shanghai Ninth People’s Hospital, Shanghai Jiao Tong University School of Medicine Ethics Committee (approval no. SH9H-2019-T279-2). In patients with “transitioning primaries,” the higher-pigmented and lower-pigmented tumor regions were independently resected and stored separately. After surgical resection, fresh tissue samples were immediately sent for scRNA-seq, and the other parts were stored in liquid nitrogen for subsequent WES and bulk mRNA-seq. Clinical data and specimens were collected with informed consent. Clinical data were obtained from the medical records system, with the access approval from the Clinical Retrospective Research Ethics Committee by the Shanghai Ninth People’s Hospital, Shanghai Jiao Tong University School of Medicine (approval no. SH9H-2021-T113-1). OS was calculated from the date of initial pathological diagnosis to the death or the last follow-up on 1 June 2023. Patients who were still alive at the last follow-up were censored. The study adhered to all relevant ethical regulations regarding research involving human participants. Detailed information of individual patients in this study has been provided in tables S1 and S5.

### Tumor samples and H&E staining

Tumor samples were retrieved, collected, and analyzed in the Department of Oral Pathology, Shanghai Ninth People’s Hospital under the standard tissue preparation protocol. In detail, harvested samples were fixed with 10% neutral buffered formalin for 12 to 48 hours, depending on the size of the samples. The samples were cut into 3- to 5-mm-thick sections and then processed with the Leica Tissue Processor (ASP300S). After the automation process was completed, the samples were embedded in paraffin. Then, the Formalin-Fixed and Paraffin-Embedded (FFPE) samples were cut into 4.0-μm slides for H&E staining. All H&E staining was carried out on the Leica ST5010 Autostainer XL.

### Immunohistochemistry

Following surgical resection, tumor specimens were fixed in 4% paraformaldehyde (PFA) and subsequently processed through dehydration, clearing, and paraffin embedding. Then, tissue sections were cut at a thickness of 4.0 μm. Sections were deparaffinized and rehydrated in a graded xylene and ethanol. For IHC analysis, heat-induced antigen retrieval was conducted with Tris-EDTA buffer (pH 9.0). Following a 30-min block of endogenous peroxidase with 3% H_2_O_2_ and a 1-hour incubation in 1% bovine serum albumin (BSA) with 0.3% Triton X-100, we then applied the primary antibodies (Key Resources) at optimal dilutions. For IHC, the DAKO AutostainerLink 48 platform (Agilent Technologies) was used according to the manufacturer’s guidelines. Signals were visualized by the DAB or the AEC+ High Sensitivity Substrate Chromogen kit (K3469, Agilent Technologies, California). Whole-slide images were acquired using an Olympus Slideview VS200 system. For quantification, the IHC score was assigned on the basis of the percentage of cells exhibiting positive staining, irrespective of the intensity. All antibodies used in this study are listed in Table 1.

**Table 1.** Antibodies used in this study, including target antigens and supplier information.

Antibodies	Source	Identifiers
Anti-NGFR	Cell Signaling	8238
Anti-AXL	Cell Signaling	8661
Anti-Phospho-HER3/ErbB3 (Tyr1289)	Cell Signaling	4791
Anti-Phospho-HER2/ErbB2 (Tyr1221/1222)	Cell Signaling	2243
Anti-HER2/ErbB2	Cell Signaling	2165
Anti-HER3/ErbB3	Cell Signaling	12708
Anti-Phospho-STAT3 (Tyr705)	Cell Signaling	9145
Anti-GP100	HUABIO	HA721719
Anti-Phospho-NF-κB p65 (S529)	HUABIO	ET1604-27
Anti-NF-κB p65	HUABIO	ER0815
Anti-STAT3	HUABIO	ET1607-38
Anti-Beta tubulin	HUABIO	ET1602-4
Anti-SOX10	HUABIO	HA721239
Anti-p75 NGF Receptor	HUABIO	ET1601-22
Anti-CD68	HUABIO	HA601115
Anti-COX2	HUABIO	ET1610-23
HRP Conjugated Goat anti-Rabbit IgG Goat Polyclonal Antibody	HUABIO	HA1001
HRP Conjugated Goat anti-Mouse IgG Goat Polyclonal Antibody	HUABIO	HA1006
Anti-GAPDH	Beyotime	AF0006
HRP Conjugated Goat anti-Rabbit IgG (H+L)	Beyotime	A0208
HRP Conjugated Goat anti-Mouse IgG (H+L)	Beyotime	A0216
FITC anti-human CD271 (NGFR) Antibody	BioLegend	345104
Anti-Melan-A	MXB Biotechnologies	MX118
Anti-SOX-10	MXB Biotechnologies	EP268
Anti-HMB45	MXB Biotechnologies	MAB-0098
Anti-S-100	DAKO	IR504
Human TNF-α Antibody	R&D	MAB210
PE Mouse Anti-Human COX-2	BD	565125
Anti-TNF-α	Boster	ba0131

### Pathological evaluation

The pigment level was assessed on the basis of the amount of the pigmentation in H&E sections. We evaluated the pigment level by measuring the area occupied by the pigmentation. The pigment score was classified into the following three categories: amelanotic, 0 to 4%; low-pigmented, 5 to 29%; and high-pigmented, 30 to 100%.

### Establishment of the MM PDX model

Tumor samples were retrieved, collected, and analyzed in the Department of Oral Pathology, Shanghai Ninth People’s Hospital under the standard tissue preparation protocol. Surgically excised tumor samples were processed within 12 hours of biopsy. Tumor tissue was minced into ~2 mm–by–2 mm fragments and implanted directly into NSG (NOD Scid Gamma Mouse) mice. Mice were anesthetized, a small skin incision (~5 mm) was made in the back of the animal, and a subcutaneous pocket was created. Tumor fragments were implanted with 100 μl of Matrigel, and the incision closed with a wound clip. Tumor size was assessed once weekly by caliper measurements (length by width by width/2). Animals were euthanized when the tumors reached 1000 mm^3^ or when necessary for animal welfare. The tumor was then reimplanted at a 1:20 ratio. The protocols for care and use of animals were approved by the Ethics Committee of Shanghai Ninth People’s Hospital (approval no. SH9H-2022-A709-SB).

### Drug treatment in the PDX model

When tumors reached 200 mm^3^, mice were randomized into treatment groups. Groups in the efficacy studies were five to six animals each to account for variability among tumors. Doxorubicin (5 mg/kg, once every 3 days) and/or afatinib [25 mg/kg,quaque die/every day (qd)] were treated in those mice. For intratumoral TNF-α injection, an equivalent of 60 ng per dose in a total 60-μl volume of PBS was injected directly into tumors. Tumor sizes were assessed twice weekly per caliper measurement, and tumor volume was estimated using the formula (length by width by width/2). Mice were euthanized after 3 weeks of treatment or when necessary for animal welfare. The chemicals and recombinant proteins used in this study are listed in Table 2.

**Table 2.** Key chemicals and recombinant proteins used in this study, including supplier details and relevant identifiers.

Reagents	Source	Identifiers
Afatinib	MCE	HY-10261
Allitinib	MCE	HY-15375
TX1-85-1	MCE	HY-100848
Doxorubicin	MCE	HY-15142
Idarubicin	MCE	HY-17381A
Daunorubicin	MCE	HY-13062A
Protein Transport Inhibitor (Containing Monensin)	BD	565125
Human IL-6	PeproTech	200-06
Human TNF-α	PeproTech	300-01A
Human IFN-γ	PeproTech	300-02
LPS	Sigma-Aldrich	L2630
PMA	Yeasen	50601ES03

### IHC staining scoring for mouse PDX tumors

For each section, five spots of a defined area (0.6 mm by 0.6 mm) were randomly selected, and protein-positive levels for each cell were counted, scoring them as 3, 2, 1, or 0 based on intensity. The IHC score for each spot was calculated using the equation: IHC score = 3*x* _% + 2*x* _% + 1*x* _% + 0*x*_%. The average IHC score of the five spots represents the overall score for each slide. We used three samples for the IHC score calculations.

### Tumor dissociation and culture of primary MM cells

Surgically excised tumor samples from patients or PDX models were placed in RPMI 1640 (Gibco, C11875500BT), containing 2% penicillin-streptomycin (Gibco, 15070063) and 1% fetal bovine serum (FBS) (Gibco, A3161002C) on ice, and transferred to the laboratory for digestion. In lab, tissue was sterilized by washing with 75% ethanol for 30 s, followed by three times of washes with RPMI 1640 (no FBS). After removing excessive pigmented debris and blood clots, tumors were minced into small pieces and incubated in type IV collagenase (2 mg/ml; Gibco, 17104019) at 37°C on a rocking platform for 30 min to 1 hour. The digestion was stopped by adding FBS. Primary melanoma cells were collected by filtering through a 70-μm cell strainer, centrifuging twice at 300*g* for 5 min, and resuspending in RPMI 1640 supplemented with 10% FBS, 1% l-glutamine (Gibco, 25030081) and 0.2% primocin (InvivoGen, ant-pm-1). The primary cells were seeded at a density of 10^5^ cells/cm^2^ and incubated at 37°C in a humidified atmosphere of 5% CO_2_ for further use. The culture medium was changed every 48 hours. Primary cells are available for sharing in accordance with the hospital’s clinical sample–sharing protocol.

### Flow cytometric analysis

MM cells were treated by IL-6 (25 ng/ml; PeproTech, 200-06) or TNF-α (PeproTech, 300-01A) for 3 days and then stained with FITC anti-human CD271 (NGFR) (BioLegend, clone ME20.4) antibody at 4°C for 30 min to determine the cell surface NGFR expression level. Flow cytometry was performed using BD FACSAria, and 10,000 cells were counted. FlowJo v.10.6.0 was used for further analysis and quantification. All antibodies used in this study are listed in Table 1.

### Immunoblotting

Cells were washed with prechilled PBS and then lysed in radioimmunoprecipitation assay (RIPA) buffer (Millipore, 20-188) containing 1× protease and phosphatase inhibitors (Thermo Fisher Scientific, 78430). The lysates were incubated for 30 min on ice, followed by ultrasonication for 1 min and centrifugation at 4°C, 12,000 rpm for 15 min. The protein concentration of the supernatant was determined using a BCA protein assay kit (Beyotime, P0009). Protein extracts were mixed with gel loading buffer (Epizyme LT103) and boiled at 90°C for 10 min. Equal amounts of protein (30 μg per lane) were separated by SDS–polyacrylamide gel electrophoresis (PAGE) on 10% gels (Bio-Rad, 1610183) and transferred to polyvinylidene difluoride (PVDF) membranes (Bio-Rad, 1620177). After blocking with 5% BSA (meilunbio, MB4219), the membranes were incubated with primary antibodies at optimal dilutions overnight at 4°C. Subsequently, membranes were washed with Tris-Buffered Saline with Tween 20 (TBST)and incubated with horseradish peroxidase (HRP)–conjugated secondary antibodies (anti-mouse, HUABIO HA1006; anti-rabbit, HUABIO HA1001). Signal detection was performed using the ECL substrate (Bio-Rad, 1705061) and imaging on a GE Amersham Imager600 system. All antibodies used in this study are listed in Table 1.

### Reverse transcription quantitative polymerase chain reaction

Total RNA was extracted using the TRIzol reagent (Invitrogen, 15596026). Next, cDNA synthesis and subsequent qPCR were performed using the HiScript III RT SuperMix (Vazyme, R323-01) and the ChamQ Universal SYBR Master Mix (Vazyme, Q341-02), respectively, on an ABI QuantStudio 7 Flex system. Gene expression was normalized to glyceraldehyde-3-phosphate dehydrogenase (GAPDH) and calculated using the 2^−ΔΔCT^ method. All primer sequences are listed in Table 3.

**Table 3.** Primer sequences used for RT-qPCR, including target gene, primer direction (forward/reverse), and sequence.

Target	Sequence
GAPDH-Human-Forward	GTCTCCTCTGACTTCAACAGCG
GAPDH-Human-Reverse	ACCACCCTGTTGCTGTAGCCAA
NGFR-Human-Forward	TCATCCCTGTCTATTGCTCCA
NGFR-Human-Reverse	TGTTCTGCTTGCAGCTGTTC
HER2-Human-Forward	TGTGTGGACCTGGATGACAAGG
HER2-Human-Reverse	CTCCGTTTCCTGCAGCAGTCT
HER3-Human-Forward	GCAACATTGATGGATTTGTG
HER3-Human-Reverse	CTCCCGTACTGTCCGGAAGAC
MITF-Human-Forward	GGCTTGATGGATCCTGCTTTGC
MITF-Human-Reverse	GAAGGTTGGCTGGACAGGAGTT
MLANA-Human-Forward	GGACAGCAAAGTGTCTCTTCAAG
MLANA-Human-Reverse	TCAGGTGTCTCGCTGGCTCTTA
MKI67-Human-Forward	GAATTGAACCTGCGGAAGAGC
MKI67-Human-Reverse	AGCGCAGGGATATTCCCTTATTTT
AURKB-Human-Forward	ATGGCCCAGAAGGAGAACTC
AURKB-Human-Reverse	TTCTCCCGAGCCAAGTACAC
AXL-Human-Reverse	GTGGGCAACCCAGGGAATATC
AXL-Human-Reverse	GTACTGTCCCGTGTCGGAAAG
EGFR-Human-Reverse	TAACAAGCTCACGCAGTTGG
EGFR-Human-Reverse	GTTGAGGGCAATGAGGACAT
MET-Human-Reverse	CTCCTTGGAAATGAGAGCTG
MET-Human-Reverse	CAGTTGAAATGGTTTGGGCTG

### Multiple IHC staining

The multiple IHC (mIHC) staining was conducted by World Advanced Science Co. Ltd. (Shanghai, China). Briefly, mIHC staining was performed with the tyramide signal amplification (TSA) IHC kit. Sections obtained from paraffin-embedded samples were dewaxed, rehydrated, and subjected to 100°C for antigen retrieval. Then, the sections were incubated with blocking antibody at room temperature for 30 min and treated with the first primary antibody overnight at 4°C, HRP-conjugated secondary antibody for 50 min at room temperature, and a TSA for 10 min. After washing in TBST buffer, the slides went through Tris-base buffer antigen retrieval using a microwave set at the maximum power mode for 15 min. The same process was repeated for the following three primary antibodies. Each slide was then treated with 4′,6-diamidino-2-phenylindole (DAPI) solution and manually coverslipped. Slides pictures were taken with a Pannoramic MIDI tissue imaging system (3D HISTECH). All antibodies used in this study are listed in Table 1.

### Quantification of mIHC staining data

To analysis the mIHC staining data in Fig. 5L, we first added circles with specific pixel dimensions (9.5 μm) to precisely quantify the proximity of cells. Each filled color within the circle represents positive staining for specific proteins: (i) Yellow dots represent SOX10+/NGFR+ staining; (ii) blue dots represent DAPI staining (for nuclei); (iii) red dots represent COX2+ macrophages; (iv) light blue dots represent CD68+ macrophages. Then, two quantification methods are used to determine the spatial relationship between different macrophage subsets and melanoma cells. All antibodies used in this study are listed in Table 1.

Quantification method one: First, the multicolor stained image is read using OpenCV or PIL and converted into a NumPy array for processing. The HoughCircles method in OpenCV is then applied to detect circular objects in the image. To differentiate colors, the image is converted into the HSV color space, allowing precise identification of circles based on their hue values. Next, the *x* and *y* coordinates of all detected circles are extracted and classified according to their color. The Euclidean distances between circles of different colors are calculated, and statistical measures such as nearest neighbor distances, mean, and SD are computed to characterize their spatial distribution. Last, the spatial organization of the color-coded objects is analyzed. The matplotlib library is used to visualize the distribution of color points, along with their nearest neighbor connections. A density plot is generated to illustrate the distribution of distances between different color-coded points, providing insights into the spatial arrangement and clustering patterns of cell types or other target structures. As shown in fig. S5F, the orange line represents the distance between SOX10+-positive cells and CD68-positive cells. The blue line represents the distance between SOX10+-positive cells and COX2+-positive cells.

Quantification method two: We first do pixel-based quantification of cell type colocalization in the multicolor immunofluorescence images as shown in fig. S5G. To determine whether COX2+ or CD68+ macrophages more closely surround SOX10+/NGFR+ melanoma cells, we will count the number of events where COX2+ macrophages are adjacent to SOX10+/NGFR+ cells and compare this to the number of events where CD68+ macrophages are adjacent to these melanoma cells.

### Macrophage polarization and coculture with MM cells

Both human monocytic THP-1 cells and PBMC-derived monocytes were used as a macrophage cell model.

THP-1 cells were maintained in RPMI 1640 containing 10% heat-inactivated FBS, 1% glutamine, and 1% penicillin-streptomycin. THP-1 monocytes were differentiated into M0 macrophages by 48 hours of incubation with phorbol 12-myristate 13-acetate (PMA; 100 ng/ml; Yeasen, 50601ES03). Macrophages were further polarized in M1 macrophages by incubation with interferon-γ (IFN-γ) (20 ng/ml; PeproTech, 300-02) and LPS (10 ng/ml; Sigma-Aldrich, L2630) for another 24 hours. In the coculture experiment, THP-1 monocytes were differentiated into M1 macrophages in a 24-well plate, whereas MMs were plated in the 24 Transwell inserts (membrane pore size of 0.4 μm; Jet Biofil, TCS021024).

PBMCs were isolated from buffy coats by Ficoll-Paque PLUS density gradient media (Cytiva, 17144002). After PBMC isolation, the cells were resuspended in serum-free RPMI 1640, the cell suspension was added in a tissue culture petri dish, and the plates were incubated in a humidified incubator with 5% CO_2_ at 37°C for 1 hour. After 1 hour, monocytes were already attached to the plates and the nonadherent cells were carefully washed with warm PBS. RMPI 1640 supplemented with macrophage colony-stimulating factor (M-CSF) (100 ng/ml; TargetMoI, TMPY-00127), 10% heat-inactivated FBS, 1% glutamine, and 1% penicillin-streptomycin was added to the plates and incubated at 5% CO_2_ at 37°C for 7 days to differentiate monocytes into nonpolarized macrophages. M1 polarization was achieved by supplementation with IFN-γ (50 ng/ml; PeproTech, 300-02) and LPS (20 ng/ml; Sigma-Aldrich, L2630) for another 24 hours. In the coculture experiment, monocytes were differentiated into M1 macrophages in a 24-well plate, whereas MMs were plated in the 24 Transwell inserts (membrane pore size of 0.4 μm; Jet Biofil, TCS021024). MM cells were prelabeled with CellTrace Far Red dye (Invitrogen, C34564) for 30 min at 37°C and then cocultured with M1 macrophages for 72 hours before flow cytometric analysis of NGFR expression of tumor cells. The chemicals and recombinant proteins used in this study are listed in Table 2.

### Two-dimensional cell viability assay

MM cells were seeded in 96-well plates (Beyotime FCP965) at a density of 10,000 cells per well and treated with TNF-α (25 ng/ml). After 3 days, the medium was replaced with 100 μl of fresh medium containing serial dilutions of the indicated drugs (afatinib, MCE HY-10261; allitinib, MCE HY-15375; tx1-85-1, MCE HY-100848; doxorubicin, MCE HY-15142) or dimethyl sulfoxide (DMSO). Following a 72-hour drug exposure, cell viability was assessed by adding 100 μl of CellTiter reagent (meilunbio, PWL111). Luminescence was measured using a Spark microplate reader (Tecan), and cell viability was calculated using the following formula

$$\text{Viable cells}\%=\frac{{\text{Luminescense}}_{\text{drug}}-{\text{Luminescense}}_{\text{blank}}}{{\text{Luminescense}}_{\text{control}}-{\text{Luminescense}}_{\text{blank}}}\times 100\%$$

The chemicals and recombinant proteins used in this study are listed in Table 2.

### Tumorsphere formation and drug assay

For three-dimensional culture of the MM cell line, 3000 cells were plated with or without IL-6 or TNF-α (25 ng/ml) per well on ultralow attachment, polystyrene 96-well plates (Corning 3474) in tumorsphere medium formulated by Dulbecco’s modified Eagle’s medium/F12 (Gibco, 11320033), B27 (Gibco, 17504044), epidermal growth factor (20 ng/ml; PeproTech, AF-100-15), basic fibroblast growth factor (10 ng/ml; PeproTech, AF-100-18B), insulin (5 μg/ml), and 0.4% BSA. The cells were incubated at 37°C and 5% CO_2_ and monitored overtime for spheroid formation. Pictures of tumorspheres formed at day 7 were taken by phase contrast light microscopy (Olympus). The number and size of spheres were counted and measured by ImageJ.

To evaluate the killing effect of the combination use of afatinib and doxorubicin on tumorspheres, 10,000 MM cells per well were seeded on ultralow attachment, 96-well plates with the aforementioned tumorsphere medium. After 72 hours, MM spheres were harvested by filtering through 40-μm nylon cell strainers and then plated back to ultralow attachment, 96-well plates with different concentrations of afatinib, doxorubicin, or DMSO control. The quantity and diameter of the remaining sephoroids were calculated after 72 hours of incubation, whereas the viability was measured by CellTiter. The synergistic effect of two drugs was calculated and visualized by the R package SynergyFinder (https://doi.org/10.1016/j.gpb.2022.01.004) (*47*). The chemicals and recombinant proteins used in this study are listed in Table 2.

### shRNA knockdown

Lentiviral particles were generated in human embryonic kidney (HEK) 293T cells by transient transfection using polyethyleneimine (PEI; Polysciences). Briefly, 4 × 10^6^ HEK293T cells were seeded in 10-cm dishes and transfected the following day with a plasmid mixture consisting of 10.8 μg of shRNA-expressing vector (encoding either scramble shRNA control or target-specific shRNAs), 4.5 μg of the packaging plasmid pΔ8.9, and 2.2 μg of the envelope plasmid pVSVG (Plasmid Vesicular Stomatitis Virus G Glycoprotein). Transfection was performed according to the manufacturer’s standard PEI protocol. Viral supernatants were harvested 48 hours posttransfection, clarified by centrifugation, and filtered through 0.45-μm low protein-binding filters (Millipore). Target cells were infected with viral supernatants supplemented with Polybrene (10 μg/ml; Sigma-Aldrich) for 12 hours, followed by replacement with fresh growth medium. Forty-eight hours after infection, cells were selected in puromycin (2.5 μg/ml; Invitrogen) until stable populations were obtained. The sequences of shRNAs used in this study were as follows: scramble shRNA: 5′-CCTAAGGTTAAGTCGCCCTCG-3′; AXL shRNA#1: 5′-GCGGTCTGC ATGAAGGAATTT-3′; AXL shRNA#2: 5′-CGAAAGAAGGAGACCCG-TTAT-3′; EGFR shRNA#1: 5′-TCTCCATAAATGCTACGAATA-3′; EGFR shRNA#2: 5′-GAATAGGTATT GGTGAATTTA-3′; ERBB2 shRNA#1: 5′-CAGTGCCAATATCCAGGAGTT-3′; ERBB2 shRNA#2: 5′-GCTGGCTGCAAGAAGATCTTT-3′; ERBB3 shRNA#1: 5′-CTTC GTCATGT TGAACTATAA-3′; ERBB3 shRNA#2: 5′-GCGATGCTGAGAACCAATA-3′; MET shRNA#1: ACAAGAUCGUCAACAAAAA; MET shRNA#2: CUACAGAAAUGGUUUCAAA.

### scRNA-seq data analysis

The procedures for sample preparation, library construction, and scRNA-seq were performed as we previously described (*46*). Fresh biopsies from individuals were acquired during the surgery and stored in the sCelLiVE Tissue Preservation Solution (Singleron) on ice. After immediate H&E staining of frozen samples during the surgery, samples were transferred to Singleron Biotechnologies (Nanjing, China) at 4°C to perform sample digestion, library construction, and scRNA-seq. For tissue disassociation and preparation, the specimens were washed three times with Hanks’ balanced salt solution (HBSS), minced into small pieces, and then digested with 3 ml of sCelLiVE Tissue Dissociation Solution (Singleron) by Singleron PythoN Tissue Dissociation System at 37°C for 30 min. The cell suspension was collected and filtered through a 40-μm sterile strainer. Afterward, the GEXSCOPE red blood cell lysis buffer (RCLB; Singleron) was added, and the mixture [cell:RCLB = 1:2 (volume ratio)] was incubated at room temperature for 5 to 8 min to remove red blood cells. The mixture was then centrifuged at 300*g* at 4°C for 5 min to remove the supernatant and suspended softly with PBS. Last, the samples were stained with Trypan blue, and the cell viability was evaluated microscopically. For library construction and sequencing, single-cell suspensions (2 × 10^5^ cells/ml) with PBS (HyClone) were loaded onto microwell chip using the Singleron Matrix Single Cell Processing System. Barcoding Beads are subsequently collected from the microwell chip, followed by reverse transcription of the mRNA captured by the barcoding beads and to obtain cDNA and PCR amplification. The amplified cDNA is then fragmented and ligated with sequencing adapters. The scRNA-seq libraries were constructed according to the protocol of the GEXSCOPE Single Cell RNA Library Kits (Singleron). Individual libraries were diluted to 4 nM, pooled, and sequenced on Illumina NovaSeq 6000 with 150–base pair (bp) paired-end reads.

The CeleScope v1.11.1 software was harnessed for scRNA-seq data analysis. The workflow encompassed the utilization of the rna mkref function to construct a reference genome index (GRCH38/hg38), comprising both genomic and transcriptomic sequences for enhanced alignment accuracy. Subsequently, the multi_rna module within CeleScope was used to calculate gene-level expression counts for individual cells. This entailed aligning raw scRNA-seq reads to the reference genome index, using unique molecular identifiers (UMIs) to ensure precise quantification and mitigate potential PCR duplicates.

Nine MM samples underwent scRNA-seq, producing individual count matrices per sample. These matrices were imported into the R programming environment, where the merge function in R combined them into a unified expression matrix. The subsequent analysis was performed in the R Seurat v4.3.0 package (*48*). Cells with a feature count (nFeature) ranging from 200 to 4000 and a mitochondrial content below 20% were retained, whereas low-quality cells were discarded. The normalization methods in Seurat were applied to the merged expression matrix, accommodating sequencing depth and technical variability. The top 2000 highly variable genes were chosen for principal components analysis (PCA) to capture significant variations across cells. To address batch effects arising from diverse experiments, we used the R Harmony v0.1.1 package (*49*), ensuring integration of data. The UMAP (Uniform Manifold Approximation and Projection) algorithm was used for the visualization of the single cells on a two-dimensional plane, enhancing the comprehension of cell clustering and relationships. For the purpose of cell clustering and identification of distinct populations within the integrated expression matrix, we used the FindNeighbors and FindClusters functions available in the R Seurat package. The FindNeighbors function was used to construct a shared nearest neighbor graph between the cells using the “KNN” approach, where the local connectivity of cells was established by identifying their nearest neighbors. Furthermore, the FindClusters function was applied to partition the cells into discrete clusters within the constructed graph at a resolution of 0.5. To uncover genes that exhibit significant expression differences between distinct cell clusters identified within our dataset, we used the FindAllMarkers function from the Seurat package. We focused on genes that demonstrated substantial changes in expression, using the criteria of a percentage of cells expressing the gene in one cluster (pct.1) greater than 0.25. In addition, we considered genes with an adjusted *P* value (adjusted *P*) below 0.05 to account for multiple testing. Furthermore, we selected genes with a log fold change (logFC) exceeding the threshold of log (1.5), highlighting genes with meaningful differential expression. By applying these criteria, we aimed to identify a subset of genes that play a pivotal role in distinguishing various cell populations within our single-cell transcriptomic dataset. The cell types were determined on the basis of the marker genes of the previous studies.

### Cell trajectory analysis

To unravel the temporal order and progression of melanoma cell states within our single-cell transcriptomic dataset, we used Monocle v2.28.0 (*50*) for cell trajectory analysis. The expression matrix of scRNA-seq data from our study was transformed into a CellDataSet object using the as.CellDataSet function. Size factors were estimated for each cell using the estimateSizeFactors function. Dispersions were estimated to account for biological variability using the estimateDispersions function. Genes with low expression were filtered out using the detectGenes function, setting a minimum expression threshold of 0.1. We retained genes that were expressed in at least 10 cells. Marker genes associated with significant changes were identified on the basis of an adjusted *P* value threshold of 0.05. These genes were selected as ordering genes for trajectory analysis. Dimensionality reduction was performed using the reduceDimension function. We reduced the data to two dimensions while retaining six dimensions for the analysis. The DDRTree method was used for dimensionality reduction, and the residual model formula was specified to include the cell identities.

### Cell-cell interaction analysis

We conducted cell-cell communication analysis using the R CellChat v1.6.1 package (*51*) and integrated Seurat framework. Specifically, we created a CellChat object by invoking the createCellChat function in the R Seurat package. The normalized scRNA-seq expression data from the Seurat object was used as the input. The CellChatDB.human database was assigned to the CellChat object using the CellChatDB.human source. We used the identifyOverExpressedGenes function to identify genes that were overexpressed within individual cell types or clusters. The identifyOverExpressedInteractions function was used to identify overexpressed interactions between different cell types. The computeCommunProb function was used to calculate the probability of communication between cells. Cell-cell communication instances were filtered using the filterCommunication function. Communication pairs with less than 10 cells in certain cell groups were excluded. The resulting communication data were obtained using the subsetCommunication function.

### Bulk RNA-seq and data processing

The surgically removed samples for bulk mRNA-seq were placed in RNAlater (Thermo Fisher Scientific, AM7020) and stored in liquid nitrogen before sending to Novogene Bioinformatics Technology Co. Ltd. (Beijing, China) to conduct mRNA-seq. Total RNA was used as input material for the RNA sample preparations. Sequencing libraries were generated using the NEBNext Ultra RNA Library Prep Kit for Illumina (NEB, USA; catalog no. E7530L) following the manufacturer’s recommendations, and index codes were added to attribute sequences to each sample. Briefly, mRNA was purified from total RNA using poly-T oligo-attached magnetic beads. Fragmentation was carried out using divalent cations under elevated temperature in NEB Next First Strand Synthesis Reaction Buffer (5X). First-strand cDNA was synthesized using a random hexamer primer and M-MuLV Reverse Transcriptase (RNase H). Second-strand cDNA synthesis was subsequently performed using DNA Polymerase I and RNase H. The remaining overhangs were converted into blunt ends via exonuclease/polymerase activities. After adenylation of 3′ ends of DNA fragments, NEB Next Adaptor with a hairpin loop structure was ligated to prepare for hybridization. To select cDNA fragments of preferentially 370 ~ 420 bp in length, the library fragments were purified with the AMPure XP system (Beverly, USA). Then, 3 μl of USER Enzyme (NEB, USA) was used with size-selected, adaptor-ligated cDNA at 37°C for 15 min followed by 5 min at 95°C before PCR. Then, PCR was performed with Phusion High-Fidelity DNA polymerase, Universal PCR primers, and Index (X) Primer. Last, PCR products were purified (AMPure XP system) and library quality was assessed on the Agilent 5400 system (Agilent, USA) and quantified by RT-qPCR (1.5 nM). The qualified libraries were pooled and sequenced on Illumina platforms with the PE150 strategy, according to the effective library concentration and data amount required.

To maintain data integrity, initial quality control was carried out on raw sequencing reads using the fastp v0.20.1 (*52*). This step encompassed the removal of low-quality reads and adapter trimming, effectively preparing the data for subsequent analysis. Following this, processed reads were aligned to the human reference genome (GRCH38/hg38) using the STAR v2.7.9a aligner (*53*) with default options. The alignment files generated by the STAR aligner underwent further processing and compressing into BAM format using samtools v1.12 (*54*). In addition, the BAM files were sorted, and cufflinks v2.2.1 was used to estimate gene abundances from the aligned reads. The output provided normalized expression values in the form of fragments per kilobase of transcript per million mapped reads (FPKM). Identification of differentially expressed genes (DEGs) was performed using the R limma v3.56.2 package (*55*).

### Unsupervised subtyping using nonnegative matrix factorization

The nonnegative matrix factorization (NMF) analysis based on gene expression profiles was used to uncover intrinsic patterns and relationships among the MM samples. The R NMF v0.26 package (*56*) was then used for NMF analysis. The expression data underwent log_2_ transformation and was filtered for gene variation (SD > 0.6). The NMF analysis was conducted across a range of ranks (2 to 6) to identify underlying latent factors in the data. Visualization of NMF results involved plotting ranks against the residual sum of squares and generating a consensus map using the consensusmap function, enabling the identification of robust sample clusters. Through this integrated approach, gene expression–based unsupervised clustering revealed valuable insights into the intrinsic patterns within the MM samples.

### WES and data processing

Samples preserved at −80°C were transported to Novogene Bioinformatics Technology Co. Ltd. (Beijing, China) via dry ice for WES. The exome sequences were efficiently enriched from 0.4 μg of genomic DNA using Agilent SureSelect Human All Exon V6 (Agilent, USA; catalog no. 5190-8864)/Agilent SureSelectXT Mouse All Exon library (Agilent, USA; catalog no. 5190-4643) according to the manufacturer’s protocol. First, genomic DNA was randomly fragmented to an average size of 180 to 280 bp by Covaris S220 (Covaris, USA). The remaining overhangs were converted into blunt ends via exonuclease polymerase activities. Second, DNA fragments were end repaired and phosphorylated, followed by A-tailing and ligation at the 3′ ends with paired-end adaptors. DNA fragments with ligated adapter molecules on both ends were selectively enriched in a PCR reaction. After PCR reaction, libraries were hybridized with liquid phase with a biotin-labeled probe, and magnetic beads with streptomycin were used to capture the exons of genes. Captured libraries were enriched in a PCR reaction to add index tags to prepare for sequencing. Products were purified using the AMPure XP system (Beverly, USA), and libraries were analyzed for size distribution by the Agilent 5400 system (Agilent, USA) and quantified by RT-qPCR (1.5 nM). The qualified libraries were pooled and sequenced on Illumina platforms with the PE150 strategy, according to the effective library concentration and data amount required.

WES was performed to elucidate the genetic variations within the human genome. Initially, a stringent quality control procedure was executed, using the fastp v0.20.1 (*52*) to rigorously preprocess the raw sequencing reads. Subsequent to the removal of low-quality reads and adapter sequences, the processed reads underwent alignment to the UCSC hg19 human reference genome using the bwa v0.7.17 aligner (*57*), yielding a coherent Sequence Alignment Map (SAM) file. After being compressed, indexed, and sorted into BAM format, PCR duplicates were eliminated to mitigate artifacts. The processed BAM file was then converted into mpileup format using samtools v1.12 mpileup, enabling per-base sequencing information retrieval. To identify somatic Single Nucleotide Variants (SNVs) and indels, VarScan v2.4.4 (*58*) was used, conducting a comparative analysis between tumor and matched normal samples. The application of VarScan’s filtering plugin, processSomatic, facilitated the identification of high-quality somatic variants with default options, ensuring result reliability. All identified variants were comprehensively annotated using ANNOVAR, providing insights into functional consequences, population frequency, and related annotations. Last, the R maftools v2.16.0 package was used to merge annotation files from all samples into a unified Mutation Annotation Format (MAF), followed by the utilization of the R maftools package for downstream analyses.

### Gene set variation analysis

To assess the combined variation in gene set activity among samples, we used gene set variation analysis (GSVA) to elucidate underlying biological pathways and processes within MM samples, using bulk gene expression data. The expression data were subjected to log_2_ transformation, and the MM sample gene set activities were computed using the R GSVA v1.48.1 package (*59*).

### Validation of the clinical significance of the transcriptomic subtypes

To demonstrate the effectiveness of our identified transcriptomic subtypes in the context of MM samples, we curated publicly available gene expression profiles from the Cancer Genome Atlas (TCGA) SKCM cohort. First, we pinpointed the distinctive signature genes of the transcriptomic subtypes by analyzing the RNA-seq data sourced from the 45 MM samples. Subsequently, these signature genes formed the basis for applying the nearest template prediction algorithm, which facilitated the prediction of subtypes for individuals from the TCGA SKCM cohort. To assess the clinical implications, we conducted a comprehensive evaluation. Using a log-rank test and Kaplan-Meier survival analysis in R survival v3.5-5 and survminer v0.4.9 packages, we determined the potential relationship between transcriptomic subtypes and OS. Notably, all statistical analyses were performed using R (v.4.3.0), adhering to a significance threshold of 0.05 to uncover relevant associations and patterns.

### Drug target mapping

In this study, we used a method to identify gene-drug pairs by mapping our chosen gene sets to a Drug-Gene Interaction Database v3.0 (*60*). We began by selecting relevant gene sets representing specific biological pathways or functions. Using the Drug-Gene Interaction Database, we mapped these gene sets to uncover potential associations between the genes in our sets and drugs with established interactions. This mapping process allowed us to systematically identify gene-drug pairs, highlighting potential therapeutic connections and interactions within the context of our study.

### Functional enrichment analysis of signature genes

For the analysis of DEGs obtained from single-cell and bulk RNA-seq data, we used a comprehensive approach to identify enriched pathways and functional associations. To achieve this, we used gene sets from the MSigDB database (*61*), encompassing known pathways, along with gene sets associated with functions such as invasion, pigmentation, and NCSC from an earlier study (*34*), as background databases. Subsequently, we used the hypergeometric test method available within the R clusterProfiler package (*62*) to assess the enrichment levels of DEGs within specific pathways or functional gene sets. Gene sets with an adjusted *P* value of <0.05 were considered statistically significant, indicating the potential involvement of DEGs in the respective pathways or functional contexts.

### Public datasets

The publicly available data used in this study encompassed diverse datasets, including bulk RNA-seq data and clinical information from the TCGA SKCM cohort, as well as scRNA-seq data from uveal melanoma, cutaneous melanoma, and acral melanoma samples. The TCGA SKCM bulk RNA-seq data and clinical details were downloaded from the UCSC Xena database (https://xenabrowser.net/datapages/), and the gene expression values were transformed into log_2_(FPKM+1). The scRNA-seq data for uveal melanoma (accession: GSE139829) and cutaneous/acral melanoma (accession: GSE215120) were obtained from the GEO NCBI database. These datasets were analyzed using UMI count matrices. To address batch effects and harmonize samples from different databases, we used the R Harmony algorithm for merging and batch effect removal in the single-cell data. Subsequent analyses followed similar methodologies as those used in the scRNA-seq analysis of MM samples.

### Calculation of gene module scores

Predefined gene modules were calculated for scRNA-seq and bulk RNA-seq data, respectively. For scRNA-seq data, the AddModuleScore function [implemented in the R Seurat package (*63*)] was used to compute the module score based on genes within the predefined gene modules; this function quantifies the average expression level of the predefined gene set for each cell, after subtracting the average expression of randomly selected control gene sets to minimize technical bias. For bulk RNA-seq data, we applied the ssGSEA method from the R GSVA package (*59*), which estimates the enrichment of the predefined gene set within the transcriptome of an individual sample by calculating a normalized enrichment score (NES).

### Statistics and reproducibility

Differences between two groups in both single-cell and bulk RNA-seq data were assessed with the Wilcoxon rank sum test. For experimental data and clinical prognostic comparisons, the Student’s *t* test was used, whereas survival analysis was conducted with the log-rank test. In all analyses, a *P* value less than 0.05 was considered statistically significant, with the following thresholds applied: *P < 0.05, **P < 0.01, ***P < 0.001, and ****P < 0.0001; n.s., not significant.
