# Supplementary material for: Tissue-specific inflammation induces cell state plasticity with oncogenic addiction in mucosal melanoma
Source: Sci Adv. 2026 Apr 1;12(14):eady4536. doi: 10.1126/sciadv.ady4536 (PMC13041752; doi:10.1126/sciadv.ady4536)
Supplement: Supplementary file 1 — Figs. S1 to S7 Legends for tables S1 to S6 [file sciadv.ady4536_sm.pdf]

Science Advances
AAAS

Supplementary Materials for

Tissue-specific inflammation induces cell state plasticity with oncogenic addiction in mucosal melanoma

Xuhui Ma *et al.*

Corresponding author: Wei Guo, guoweicn@sjtu.edu.cn; Hanlin Zeng, hanlin.zeng@shsmu.edu.cn

Sci. Adv. **12**, eady4536 (2026)
DOI: 10.1126/sciadv.ady4536

The PDF file includes:

Figs. S1 to S7
Legends for tables S1 to S6

Other Supplementary Material for this manuscript includes the following:

Tables S1 to S6

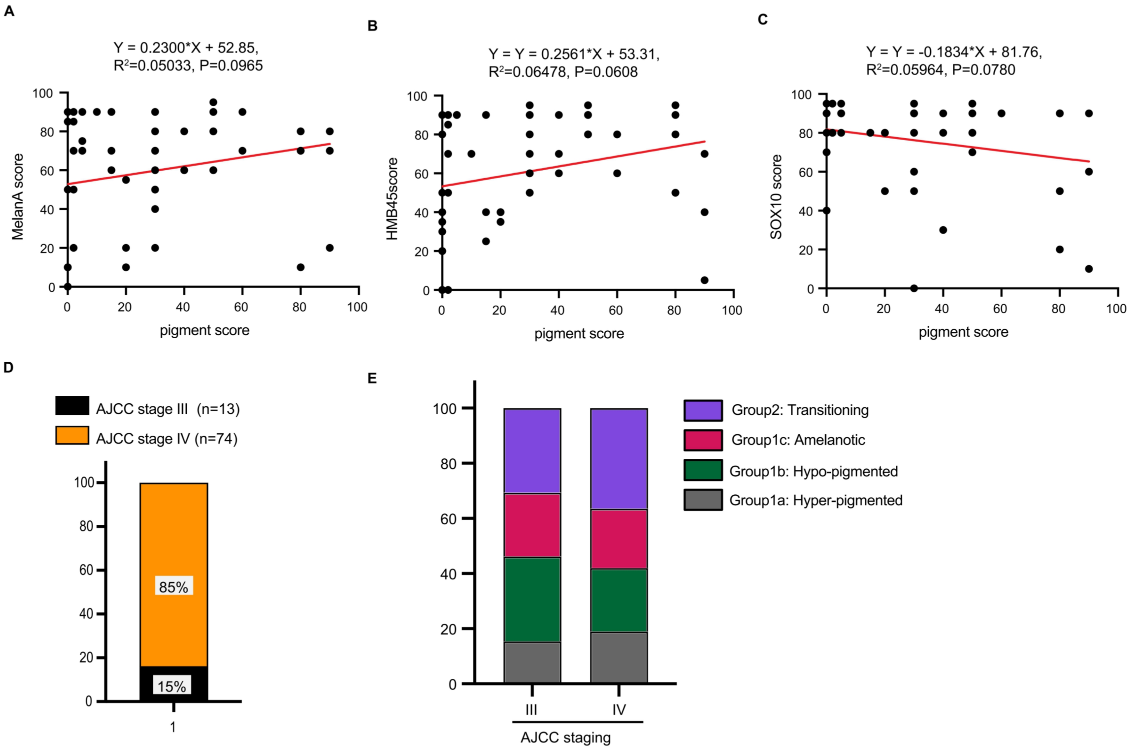

Fig. S1. Correlation of pigment score, melanocyte marker genes expression and prognosis in mucosal melanoma patients, related to Fig. 1. (A-C) Correlation of pigment score with melanA (**A**) HMB45 (**B**) and SOX10 (**C**) expression. Pigment score was defined using Hematoxylin and Eosin (H&E) staining, while melanA, HMB45, and SOX10 scores were determined through antibody-based IHC staining. **(D)** Distribution of mucosal melanoma samples with different melanoma stages (based on AJCC Cancer Staging Manual). **(E)** Distribution of mucosal melanoma samples with varying pigmentation levels across different AJCC stages.

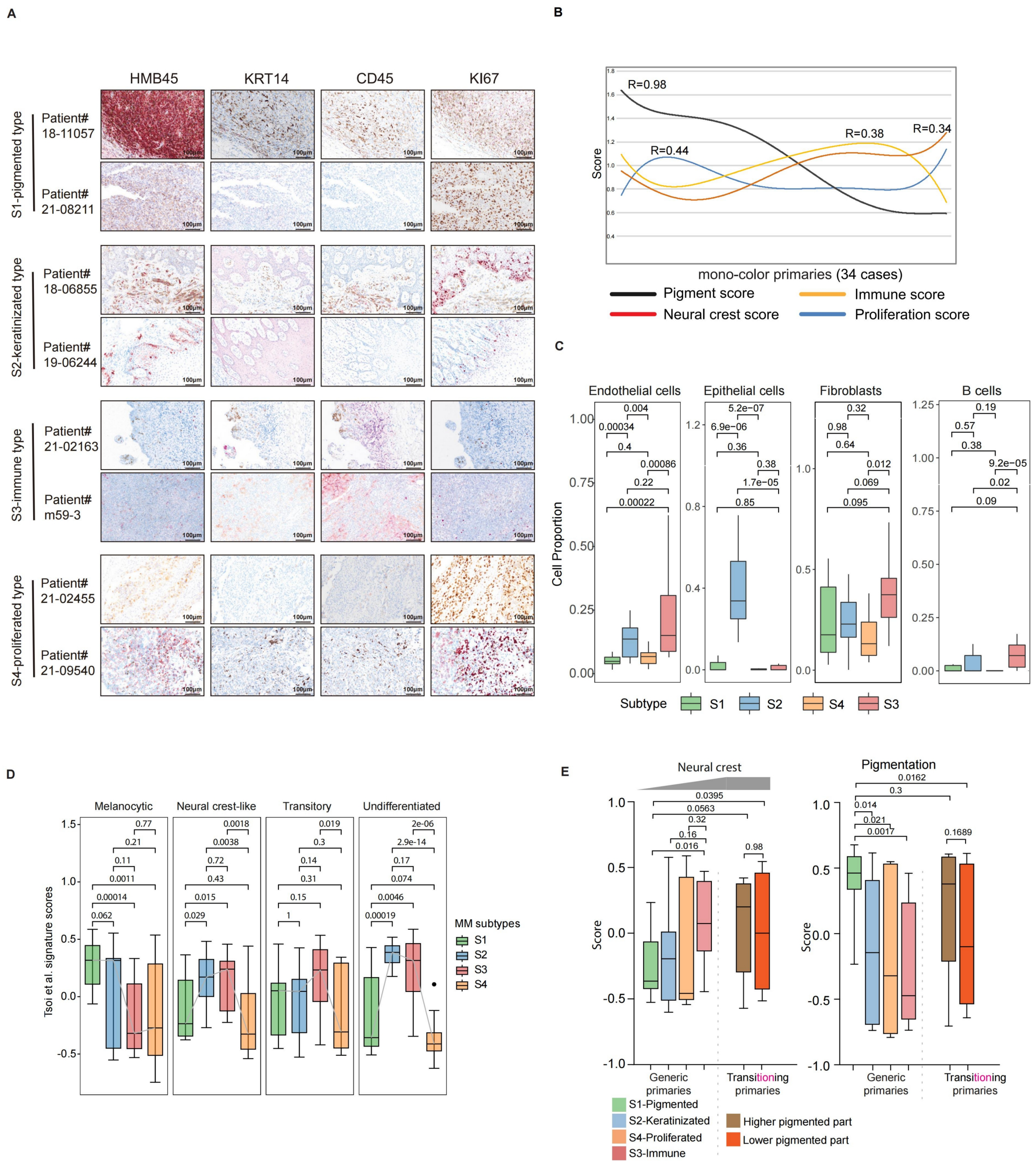

Fig. S2. Defining four mucosal melanoma subtypes, related to Fig. 2 (A) IHC staining for HMB45, CD45, Ki67, and KRT14 in tumor samples representing the S1–S4 subtypes of mucosal melanoma. All slides utilized AP (alkaline phosphatase) red chromogen for staining, except for HMB45 and Ki67 in Patient S1-# 21-08211 and S4-# 21-02455, which were stained using DAB (3,3'-diaminobenzidine) brown chromogen for enhanced visualization. (**B**) ssGSEA score analysis evaluating the association between pigment score, neural crest score, immune score, and proliferation score of patients with generic primaries based on bulk mRNA-seq data.

Gene sets for the indicated pathways are listed in table S4. **(C)** Predicted proportion of microenvironmental cells in each mucosal melanoma subtype by analyzing bulk mRNA-seq data using CIBERSORT. P values are indicated on top of the bars (two-tailed t-test). **(D)** Scoring mucosal melanoma subtypes using four distinct gene signature sets from Tsoi et al. paper (*28*), including Melanocytic, Neural Crest-like, Transitory, and Undifferentiated gene sets. **(E)** Enrichment score analysis of neural crest signature and pigment signature in transitioning primaries and generic primaries based on correlated bulk mRNA-seq data. P values are labeled on top of the bars (two-tailed t test). Box and whisker plot represents mean, 10th and 90th percentiles, minimum and maximum values of all correlated scores.

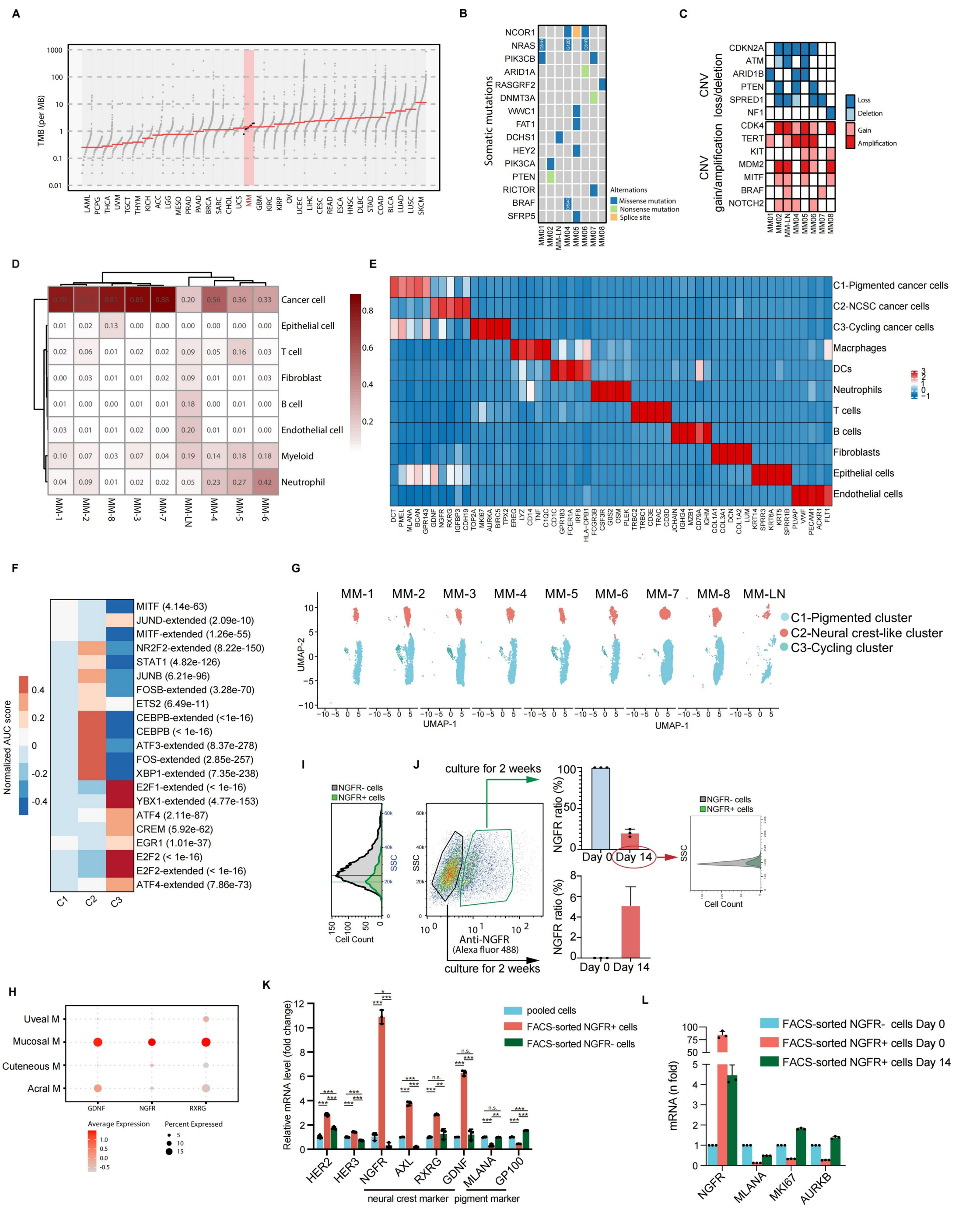

Fig. S3. Identification of three district cancer cell states through single-cell RNA sequencing of mucosal melanoma samples, related to Fig. 3. **(A)** Tumor mutation burden of mucosal melanoma compared with other cancer types from TCGA whole-exome sequencing (WES) data. (**B)** Oncoplot of mutated genes in the mucosal melanoma WES cohort. (**C)** Oncoplot of recurrent genes with copy number changes in the mucosal melanoma WES cohort. (**D)** Composition of all cell clusters from single-cell RNA-seq of mucosal melanoma. (**E)** Heatmap of representative gene

expression across cell types. Cancer cells were subclustered into Pigmented, Neural Crest Stem-like (NCSC), and Cycling states. **(F)** Heatmap of transcription factor activation in three cancer cell clusters. (**G**) Proportions of cancer subclusters in each mucosal melanoma sample (n=9). (**H**) Expression of neural crest markers (GDNF, NGFR, RXRG) compared across melanoma subtypes by integrative single-cell analysis. (**I)** Fluorescence-activated cell sorting (FACS) analysis of side scatter (SSC) score difference between NGFR+ and NGFR- cells. **(J)** FACS analysis of SSC score between NGFR+ cell ratio changes after 14 days of culturing the pre-sorted NGFR+ mucosal melanoma cells and NGFR- mucosal melanoma cells. Represented FACS result shows NGFR status before FACS sort (middle), and bar graph of NGFR+ cell ratio changes after 14 days of culture (right). (**K)** RT-QPCR neural crest– and pigment-associated genes in FACS-sorted $NGFR^+$ versus $NGFR^-$ OMM-1 cells; pooled unsorted cells served as control. (**L)** Comparison of gene expression by RT-QPCR between FAC-sorted NGFR- mucosal melanoma cells and FACS-sorted NGFR+ mucosal melanoma cells cultured on Day 0 and Day 14 (n=3). Statistical significance by Student's t-test (*$p < 0.05$, **$p < 0.01$, ***$p < 0.001$; n.s., not significant).

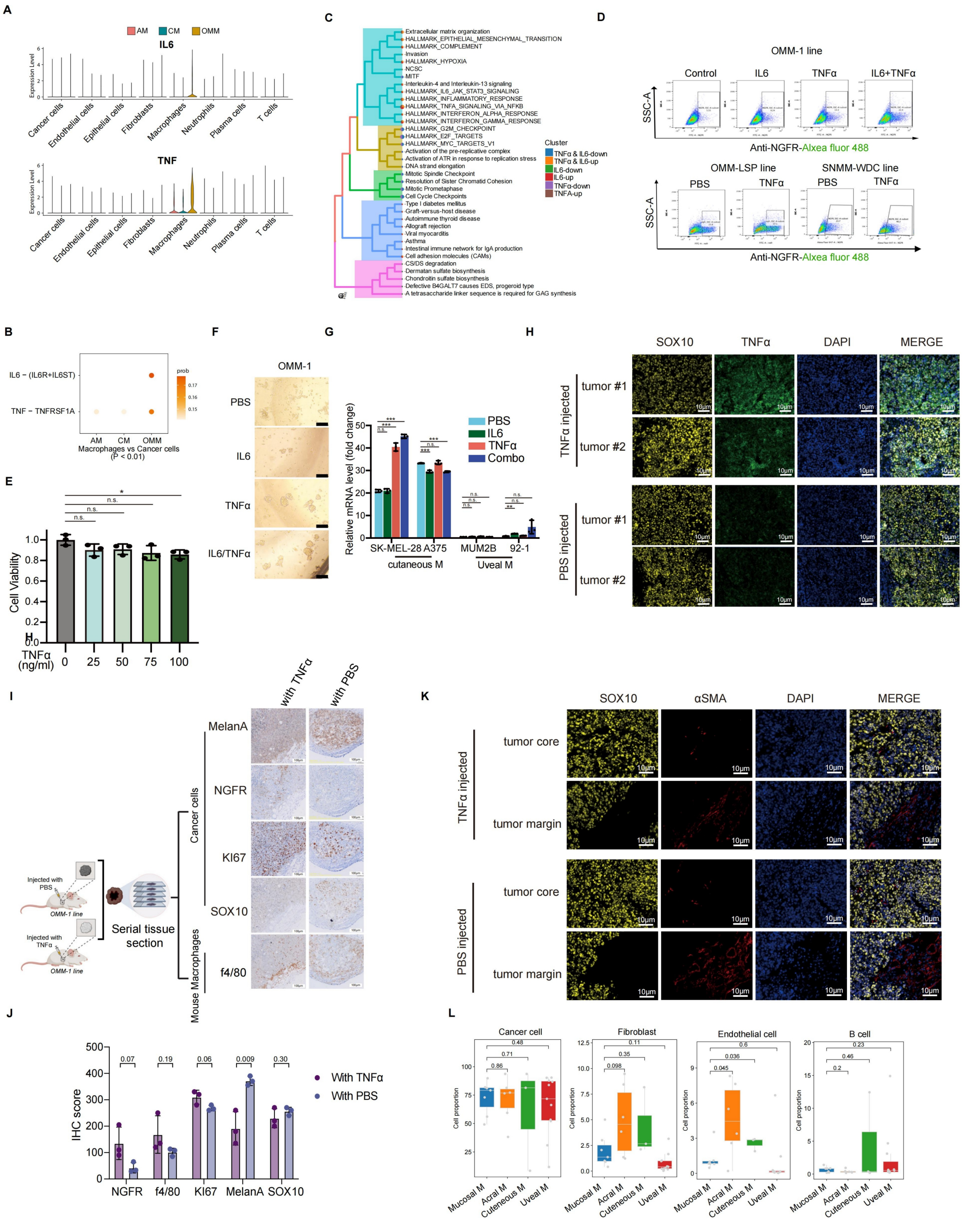

Fig. S4. TNFα induces neural crest-like cancer cell state in mucosal melanoma, related to Fig. 4. (**A**) Violin plots comparing IL6 and TNF expression levels across different cell types in acral melanoma (AM), cutaneous melanoma (CM), and oral mucosal melanoma (OMM) based on scRNA-seq data. (**B**) Strength of IL6- and TNF-specific ligand–receptor interactions between cancer cells and macrophages across AM, CM, and OMM. **(C)** Heatmap of differentially expressed genes (DEGs)

after 72 hrs TNFα, IL6, or combined treatment in OMM-1 cells. **(D)** FACS of NGFR+ cell proportions in OMM-1 cells, OMM_LSP cells (oral mucosal melanoma cells) and SNMM_WDC cells untreated or treated with TNFα, IL6, or TNFα/IL6 combination for 72 hrs. (**E)** Cell viability of OMM-1 after 72 hrs TNFα treatment at 25–100 ng/ml; PBS as control. **(F)** Representative spheroid formation images of OMM-1 cells untreated or treated with TNFα, IL6, or combination for 7 days. Scale bar, 500 μm. **(G)** RT-QPCR of NGFR mRNA in cutaneous melanoma (SKMEL-28, A375) and uveal melanoma (MUM2B, 92-1) cells after 72 hrs IL6, TNFα, or combination treatment (50 ng/ml). **(H)** Multicolor IHC of TNFα and SOX10 in OMM-1 PDX tumors injected with PBS or TNFα. (**I-J)** Immunohistochemistry staining **(I)** and quantification **(J)** of MelanA, NGFR, SOX10, and F4/80 in OMM-1 PDX tumors after PBS or TNFα injection using serial tissue sections. (**K)** Multicolor IHC staining of α-SMA and SOX10 in tumors from the OMM-1 PDX model following local injection of either PBS or recombinant TNFα. (**L)** Bar graph of cancer and microenvironmental cell proportions in mucosal melanoma compared with acral melanoma, cutaneous melanoma, and uveal melanoma. Statistical significance by Student's t-test (*$p < 0.05$; **$p < 0.01$; ***$p < 0.001$; n.s., not significant).

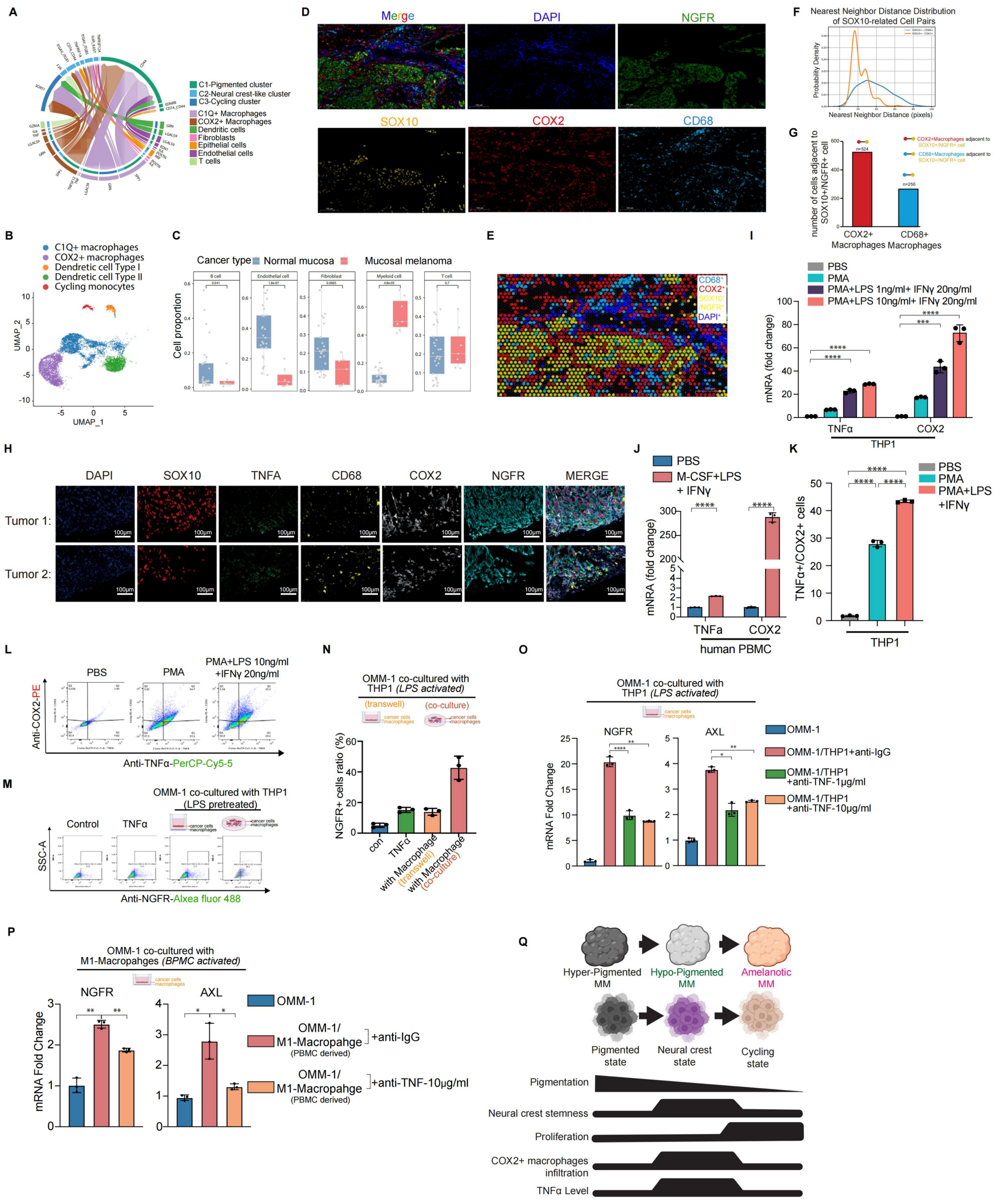

Fig. S5. TNFα-secreting COX2+ macrophages are induced during mucosal inflammation, related to Fig. 5. (A) Cell-cell interactions between the three cancer cell clusters and all microenvironmental cells from mucosal melanoma single-cell RNA-seq data; arrows indicate ligand→receptor interactions, populations are color-coded. **(B)** UMAP of monocyte-derived cell clusters through integrative analysis of single-cell mRNA sequencing data from healthy mucosa, periodontitis, and mucosal melanoma. **(C)** Bar plot comparing microenvironmental cell proportions

in healthy mucosa versus mucosal melanoma. (**D)** Multi-color immunofluorescence (IF) staining of mucosal melanoma tissue sections, including individual single-label images for each marker. **(E)** Pixel-based quantification of cell co-localization (9.5 μM pixels): yellow, SOX10+/NGFR+ cancer cells; blue, DAPI; red, COX2+ macrophages; light blue, CD68+ macrophages. (**F**) Quantification of the distances between SOX10+/NGFR+ melanoma cells and macrophage subsets. The orange line represents the distance between SOX10+/NGFR+ melanoma cells and CD68+ macrophages, while the light blue line represents the distance between SOX10+/NGFR+ melanoma cells and COX2+ macrophages. **(G)** Quantification of macrophage subsets adjacent to SOX10+/NGFR+ melanoma cells. The red bar represents the number of instances where COX2+ macrophages are adjacent to SOX10+/NGFR+ melanoma cells, while the light blue bar represents the number of instances where CD68+ macrophages are adjacent to these melanoma cells. **(H)** Multiplex IF staining of CD68, COX2 , SOX10, NGFR, TNFα and DAPI. Representative images of two tumors from S3 mucosal melanoma subtype are shown. **(I-J)** RT-QPCR validation of increased TNFα and COX2 mRNA levels after induction of M1 macrophage subtype transition induced by PMA+LPS+IFNγ combinations in THP1 cells (I) and human PBMC cells (J). **(K-L)** FACS analysis of TNFα and COX2 co-expression after induction of M1 macrophage subtype transition induced by PMA+LPS+IFNγ combinations in THP1 cells. **(M-N)** FACS analysis of NGFR+ OMM-1 cells' proportion after treatment with PBS (control), TNFα, and co-culturing with LPS-activated THP1 cells (n=3). "Transwell" represents LPS-activated THP1 cells cultured in the upper chamber, and cancer cells cultured in the lower chamber. "Co-culture" represents cancer cells (prelabelled with CellTrace Far Red Dye) and LPS-activated THP1 cells in the same culture dish, followed by FACS analysis of NGFR+ cancer cells proportion. (**O)** RT-QPCR analysis of NGFR and AXL expression levels in OMM-1 cells co-cultured with THP1 macrophages, with or without TNFα blocking antibody. **(P)** RT-QPCR analysis of NGFR and AXL expression levels in OMM-1 cells co-cultured with M1 macrophages derived from human PBMC of healthy donor, with or without TNFα blocking antibody. **(Q)**

Schematic illustrating the transition of cancer cell mRNA expression programs and microenvironmental cell compositions during mucosal melanoma depigmentation. Statistical significance: Student's t-test (*$p<0.05$, **$p<0.01$, ***$p<0.001$, n.s., not significant).

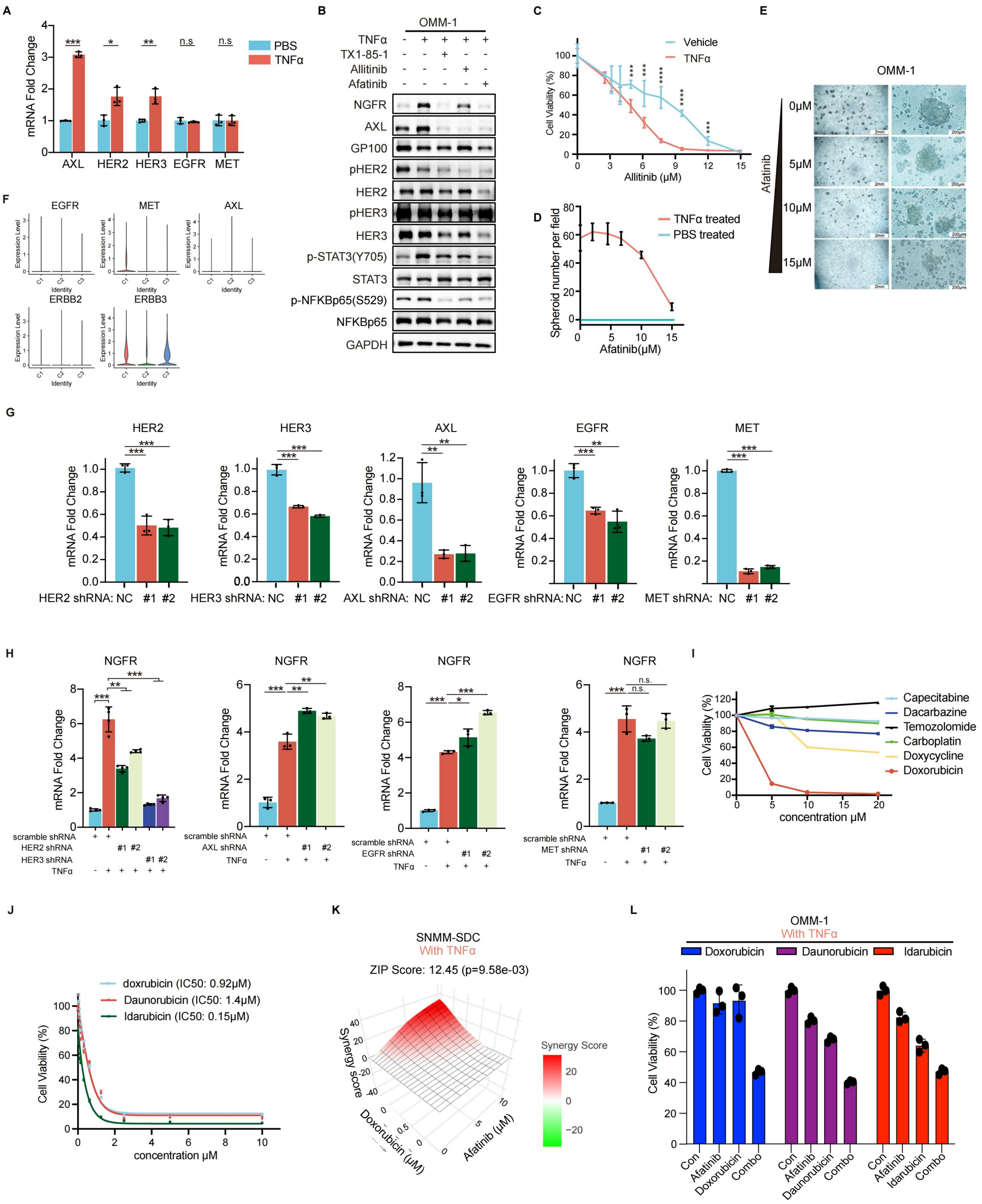

Fig. S6. COX2+ macrophages-induced neural crest-like mucosal melanoma Cancer Cells Are Addicted to HER2 and HER3, related to Fig. 6. (A) Bar graph of HER2, HER3, AXL, EGFR, and MET mRNA changes in OMM-1 cells after 72 hrs TNFα treatment (n=3). **(B)** Western blot of HER2/HER3 signaling with TNFα ± HER inhibitors (Afatinib, Allitinib, TX1-85-1). in OMM-1 cells. (**C)** Dose-response curves showing increased sensitivity to Allitinib in OMM-1 line with TNFα-induced

dedifferentiation for 72 hrs (n=3). TNFα: 25ng/ml. (**D)** Dose-response curves demonstrating the inhibition of spheroid formation by Afatinib treatment for 6 days in OMM-1 cells with or without TNFα (n=3). **(E)** Representative images of spheroid formation by Afatinib treatment for 6 days in OMM-1 cells with or without TNFα. **(F)** mRNA expression patterns of *ERBB2*, *ERBB3*, *EGFR*, *MET*, and *AXL* across the three mucosal melanoma clusters identified by scRNA-seq analysis (C1: pigmented; C2: neural crest–like; C3: cycling). **(G)** RT-QPCR validation of shRNA knockdown efficiency for *ERBB2*, *ERBB3*, *AXL*, *EGFR*, and *MET* in OMM-1 cells. (**H**) RT-QPCR analysis of the effect of shRNA-mediated knockdown of *ERBB2*, *ERBB3*, *AXL*, *EGFR*, and *MET* on TNFα-induced *NGFR* expression in OMM-1 cells (50 ng/ml, 72 hrs). (**I**) Cell viability of OMM-1 cells treated with chemotherapeutic agents, including capecitabine, dacarbazine, temozolomide, carboplatin, doxycycline, and doxorubicin, at serial concentrations for 72 hrs (n = 3). (**J**) Cell viability and IC50 of OMM-1 cells treated with anthracyclines (doxorubicin, daunorubicin, and idarubicin) for 72 hrs (n = 3). **(K)** Synergistic score of combined treatment of Afatinib and Doxorubicin in TNFα treated SNMM-SDC cells. **(L)** Cell viability after treating TNFα-incubated OMM-1 line with Afatinib, chemotherapeutic drugs, or their combinations for 72 hrs (n=3). Doxorubicin: 1μM; Daunorubicin: 500nM; Idarubicin: 125nM; Afatinib: 5μM; TNFα:25ng/ml. Statistical significance: Student's t-test (*p<0.05, **p<0.01, ***p<0.001, n.s., not significant).

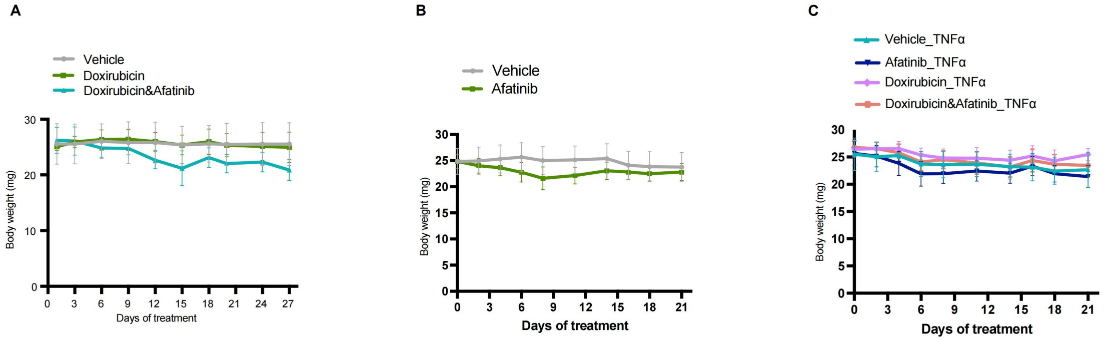

Fig. S7. In vivo validation of a pan-HER inhibitor combined with chemotherapy in a mucosal melanoma PDX model, related to Fig. 7. (A-C) Evaluation of mice body weight change by treatment with Afatinib, Doxorubicin, or their combination in the OMM-1 PDX model without **(A-B)**, or with **(C)** TNFα injection into the tumors.

table S1. Immunohistochemical (IHC) assessment of pigmentation score and expression of pigmentation markers in patients with mucosal melanoma.

table S2. All statistical analysis and differentially expressed gene list for each enriched pathway associated with Fig.2B.

table S3. Single-sample gene set enrichment analysis (ssGSEA) scores for individual mucosal melanoma samples derived from bulk mRNA sequencing data.

table S4. Gene lists of key pathways used for bulk and single-cell mRNA sequencing data analysis.

table S5. Detailed clinical information for the 9 mucosal melanoma patients with tumor scRNA sequencing.

table S6. Detailed log2FC and adjusted p-values associated with Fig.3E.
